# The Internal‐External Synergy of Self‐Reconstructed C/NiFeOOH/SeO_4_^2−^ for Efficient and Stable Seawater Electrolysis

**DOI:** 10.1002/advs.202523396

**Published:** 2026-01-27

**Authors:** Zhe Sun, Yitong Yin, Siyuan Liu, Bo Liao, Bing He, Zhaojie Wang, Xiaoqing Lu, Xingheng Zhang

**Affiliations:** ^1^ Department of Chemical and Petroleum Engineering University of Calgary Calgary Alberta Canada; ^2^ Shandong Key Laboratory of Intelligent Energy Materials School of Materials Science and Engineering China University of Petroleum (East China) Qingdao P. R. China; ^3^ School of Petroleum Engineering China University of Petroleum (East China) Qingdao P. R. China

**Keywords:** internal‐external synergy, oxygen affinity, oxygen evolution reaction, seawater electrolysis, self‐reconstructed

## Abstract

Renewable energy driven seawater electrolysis represents a promising approach for generating hydrogen. However, the development of seawater electrolysis is hindered by Cl–induced corrosion and competitive reaction. Transition metal selenides (TMSes), as promising anode catalysts, is impeded by reconstruction phenomena during the oxygen evolution reaction (OER), which leads to inefficient adsorption of OER intermediates and poor stability. Herein, an internal‐external synergy strategy of self‐reconstructed C/NiFeOOH/SeO_4_^2−^ was proposed to construct efficient and stable seawater electrolysis. The introduction of C accelerates the reconstruction process of C@FeNi_2_Se_4_ to C/NiFeOOH/SeO_4_^2−^. Internal C and external SeO_4_^2−^ jointly supply electrons to NiFeOOH to stabilize the valence state, forming a bidirectional electron‐stable structure. The C/NiFeOOH/SeO_4_^2−^ exhibits a low overpotential of 223 and 310 mV at 10 and 100 mA cm^−2^ in 1 M KOH + 0.5 M NaCl, along with exceptional stability 150 h at 400 mA cm^−2^. Experimental and density functional theory calculations reveal that the SeO_4_^2−^ layer enhances the resistance to Cl^−^ through electrostatic repulsion, and C increases the dissolution potential of NiFeOOH through strong electronic interaction with NiFeOOH, inhibiting the dissolution of metal sites. Meanwhile, SeO_4_^2−^ weakens the oxygen affinity of NiFeOOH and reduces the reaction energy barrier, while C ensures the rapid supply of electrons.

## Graphical abstract

This work presents an internal‐external synergy strategy where a C@FeNi_2_Se_4_ core‐shell precursor reconstructes in to a C/FeNi_2_Se_4_/SeO_4_^2−^ architecture during OER. The inner C stabilizes active sites while the outer SeO_4_^2−^ layer repels Cl^−^, achieving efficient and stable seawater oxidation for over 150 h at 400 mA cm^−2^.

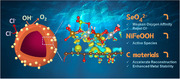

## 1 Introduction

The excessive exploitation and consumption of fossil fuels have intensified global energy and environmental challenges. Water electrolysis offers a promising pathway for converting renewable energy resources (e.g., solar and wind energy) into clean hydrogen (H_2_) fuel [1, 2]. However, traditional water electrolysis technologies rely on high‐purity deionized water (resistivity ≥18.2 MΩ cm) or alkaline electrolytes with stringent purity requirement [3, 4]. Direct utilization of seawater, constituting approximately 96.5% of Earth's total water reserves, as an electrolyte feedstock could mitigate potential strain on increasingly scarce freshwater resources driven by population growth and pollution within a growing hydrogen economy [5, 6, 7]. Alkaline seawater electrolysis represents a technologically promising approach, leveraging seawater's vast abundance [8, 9]. However, the complex composition of seawater, particularly the presence of high‐concentration chloride ions (Cl^−^), poses significant challenges to electrocatalyst stability [10, 11]. The Chlorine evolution reaction (ClER) competes with the oxygen evolution reaction (OER) and induces severe corrosion of the electrolyzer [12, 13]. The thermodynamics and kinetics of OER and ClER reveal that alkaline conditions provide a substantially wider operational overpotential window (∼0.48 V) on anode materials [14, 15]. The design of electrocatalysts that simultaneously resist Cl^−^‐induced corrosion and maintain high OER activity is therefore imperative for advancing seawater electrolysis technology [16, 17].

To address the mentioned challenges, a series of seawater electrocatalysts have been developed using inexpensive and widely available non‐precious metals, including metal hydroxides, phosphides, nitrides, and more [18, 19, 20, 21, 22]. Thereinto, transition metal selenides (TMSes) have been demonstrated as promising anode electrocatalysts for seawater electrolysis, exhibiting high OER activity due to their tunable band gap and rapid charge transfer characteristics. Notably, TMSes undergo in situ surface reconstruction into metal oxyhydroxide (MOOH), which serve as the active phase during OER process [23, 24]. However, the structure‐property relationship governing surface reconstruction under the working conditions remains insufficiently elucidated [25, 26, 27]. In the reconstructed TMSes, transition metal is generally regarded as the active site to interact with oxygen‐containing species, because the electronic structure of the metal center plays a critical role in the OER activity [28, 29, 30]. Conversely, Se also plays a vital role in regulating the electronic structure of adjacent cationic active sites [31, 32]. Therefore, the interaction between the reconstructed MOOH and Se is crucial for enhancing the activity of the catalyst [33, 34]. Furthermore, the MOOH active species suffers from intrinsic instability due to its high oxidation state [35, 36]. During the OER process, the metal active sites are prone to dissolution at continuous oxidation. Consequently, the fundamental challenge for TMSes lies in controlling reconstruction dynamics to enhance activity, stability, and chloride tolerance [37, 38]. To address this issue, it is essential to employ structural and electronic engineering strategies to design efficient surface‐reconstructed TMSes catalysts capable of achieving efficient and stable OER in seawater electrolysis [39, 40].

Herein, we proposed an internal‐external synergy of self‐reconstructed C/NiFeOOH/SeO_4_^2−^ achieves efficient and stable seawater electrolysis. The introduction of C sphere accelerated the surface reconstruction of C@FeNi_2_Se_4_ during the OER process, in which C@FeNi_2_Se_4_ was in situ transformed into C/NiFeOOH/SeO_4_^2−^ structure. As a result, the C/NiFeOOH/SeO_4_^2−^ achieves a low overpotential of 223 and 310 mV at 10 and 100 mA cm^−2^, respectively, in 1 M KOH + 0.5 M NaCl. It also exhibits exceptional stability, maintaining performance for over 150 h at 400 mA cm^−^^2^. Furthermore, under industrially relevant conditions of 6 M KOH + seawater at 60 °C, C/NiFeOOH/SeO_4_^2−^ demonstrates outstanding durability, showing no significant degradation in oxygen evolution reaction (OER) activity after 100 h of operation, thereby highlighting its potential for industrial applications. Experimental and density functional theory (DFT) calculations reveal that the adsorption of SeO_4_^2−^ on NiFeOOH surface weakens the oxyphilic property of surface and reduces the OER energy barriers, enhancing the OER catalytic activity. Meanwhile, the SeO_4_^2–^derived oxygen‐containing anion layer on the catalyst surface effectively repels Cl^−^ via electrostatic repulsion, thereby significantly enhancing the corrosion resistance of the catalyst. Furthermore, the electronic interaction between C and NiFeOOH increases the dissolution energy barrier of metal sites, which is conductive to the stability of metal sites. This work offers a novel strategy for modulating surface reconstruction in TMSes, providing valuable insights for the design of highly efficient and stable OER catalysts for seawater electrolysis.

## 2 Results and Discussion

### 2.1 Preparation and Structural Characterization

**FIGURE 1:**
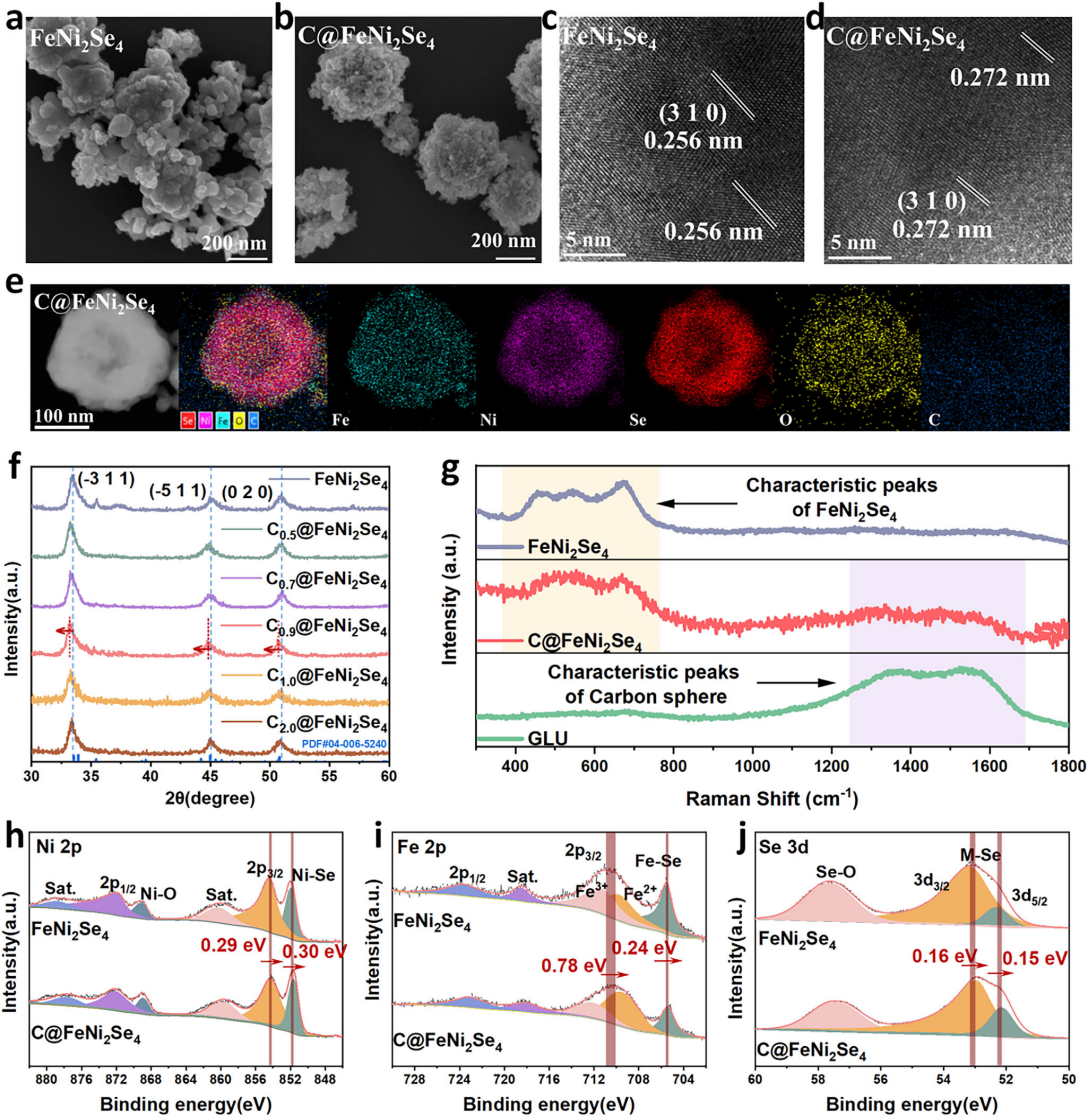
SEM images of (a) FeNi_2_Se_4_ and (b) C@FeNi_2_Se_4_. HRTEM images of (c) FeNi_2_Se_4_ and (d) C@FeNi_2_Se_4_. Corresponding elemental mappings of (e) C@FeNi_2_Se_4_. (f) XRD patterns of FeNi_2_Se_4_, C_0.5_@FeNi_2_Se_4_, C_0.7_@FeNi_2_Se_4_, C_0.9_@FeNi_2_Se_4_, C_1.0_@FeNi_2_Se_4_, and C_2.0_@FeNi_2_Se_4_ (g) Raman spectrums of FeNi_2_Se_4_, C@FeNi_2_Se_4_, and GLU samples. High‐Resolution XPS spectra of (h) Ni 2p, (i) Fe 2p, and (j) Se 3d for FeNi_2_Se_4_ and C@FeNi_2_Se_4_.

**FIGURE 2:**
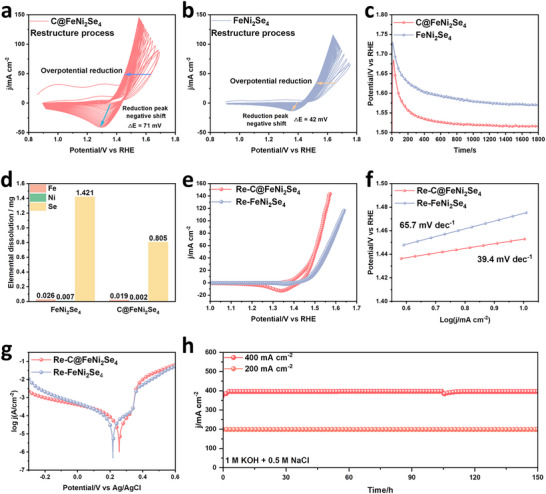
In situ CV electrochemical reconstruction process of (a) C@FeNi_2_Se_4_ and (b) FeNi_2_Se_4_ in 1 M KOH + 0.5 M NaCl. (c) Chronopotentiometric curves of FeNi_2_Se_4_ and C@FeNi_2_Se_4_ at 100 mA cm^−2^ in 1 M KOH + 0.5 M NaCl. (d) The amount of elemental dissolution of FeNi_2_Se_4_ and C@FeNi_2_Se_4_ in the reconstruction process. The (e) CV curves and (f) Tafel slope for Re‐FeNi_2_Se_4_ and Re‐C@FeNi_2_Se_4_ after reconstruction. (g) Corrosion polarization plots of Re‐FeNi_2_Se_4_ and Re‐C@FeNi_2_Se_4_ in 1 M KOH + 0.5 M NaCl. (h) Durability test of Re‐FeNi_2_Se_4_ and Re‐C@FeNi_2_Se_4_ at 200 and 400 mA cm^−2^.

As shown in Figure 1a and Figure S1, the FeNi_2_Se_4_ nanoparticles were synthesized by a simple one‐step hydrothermal synthesis method at 150°C. When glucose is introduced into the synthetic system, the core‐shell structured C@FeNi_2_Se_4_ was formed. In brief, glucose undergoes a hydrothermal carbonization process to form carbon spheres, then, FeNi_2_Se_4_ nanoparticles gradually accumulate on the surface of the carbon spheres, forming a loose FeNi_2_Se_4_ shell layer (Figure 2b). High‐magnification transmission electron microscopy (HRTEM) image reveals a typical lattice fringes with a d‐spacing of 0.256 nm, which matches well with the (3 1 0) planes of monoclinic FeNi_2_Se_4_ (Figure 1c). Notably, as shown in Figure 1d, the lattice spacing of (3 1 0) planes of C@FeNi_2_Se_4_ was expanded to 0.272 nm as compared to that of FeNi_2_Se_4_. The core‐shell structure of C@FeNi_2_Se_4_ was indicated by elemental mapping images in Figure 1e. The elements Fe, Ni, and Se are mainly present in the outer layer, while C and O are mainly distributed in the inner layer, forming a core‐shell structure in which FeNi_2_Se_4_ nanoparticles wrap the C spheres. To verify the structure of samples, we prepared a series of C@FeNi_2_Se_4_ samples experimentally. As displayed in Figure 1f, powder XRD patterns of FeNi_2_Se_4_ conform to the crystal of monoclinic FeNi_2_Se_4_ (PDF#04‐006‐5240). Particularly, the main diffraction peaks of C@FeNi_2_Se_4_ shift to small angles, revealing the increase of lattice spacing induced by C spheres, which is also consistent with the HRTEM result. The lattice expansion of FeNi_2_Se_4_ is caused by the lattice mismatch between amorphous C and crystalline FeNi_2_Se_4_ [41]. For further to comparison, C spheres were obtained by conducting the same synthesis process under the same conditions without adding Ni(NO_3_)_2_·6H_2_O and Fe(NO_3_)_3_·9H_2_O during the synthesis process. The SEM images and corresponding element mapping results indicate that glucose is transformed into C spheres at 150°C (Figure S2).

The Raman spectrum was carried out to further to explore the surface structure of samples (Figure 1g). The characteristic peaks between 1200–1600 cm^−1^ in the Raman spectra of C spheres attributed to the unique D and G peaks of carbon materials, which proves the formation of C spheres during the preparation process. In addition, peaks at 400–800 cm^−1^ are assigned to the characteristic peaks of FeNi_2_Se_4_ [42]. The C@FeNi_2_Se_4_ exhibits the characteristic peaks of selenide similar to FeNi_2_Se_4_, indicating the presence of FeNi_2_Se_4_ structure on the catalyst surface. However, there is a weak peak of C material in the Raman spectrum of C@FeNi_2_Se_4_ because the signal of the C sphere as the core is blocked by FeNi_2_Se_4_, which also proves the core‐shell structure of C@FeNi_2_Se_4._ To further investigate the electronic structure, X‐ray photoelectron spectroscopy (XPS) was performed. The Ni 2p spectrum of C@FeNi_2_Se_4_ exhibits peaks at 854.16 and 872.12 eV, corresponding to the 2p 3/2 and 2p 1/2 states of Ni, while peak at 851.69 eV is attributed to Ni‐Se (Figure 1h). Compared to FeNi_2_Se_4_, these peaks shift negatively by 0.3 eV in C@FeNi_2_Se_4_, indicating interfacial charge redistribution induced by C spheres. Similarly, the Fe 2p spectrum shows characteristic peaks at 710.05, 723.22, and 705.34 eV, corresponding to the 2p 3/2, 2p 1/2, and Fe‐Se in C@FeNi_2_Se_4_ (Figure 1i). These peaks shift to the lower binding energy in C@FeNi_2_Se_4_ relative to FeNi_2_Se_4_. Meanwhile, for the Se 3d spectrum, two peaks located at 52.13 and 52.98 eV could be attributed to Se 3d 5/2 and Se 3d 3/2, which are consistent with the signals of M‐Se (Figure 1j) [43]. The O1s spectrum of C@FeNi_2_Se_4_ also shows a similar phenomenon (Figure S3a). These peaks of C@FeNi_2_Se_4_ shift to the lower binding energy compared to FeNi_2_Se_4_. Therefore, the shift of the characteristic peaks of Fe, Ni, and Se elements indicates that the electrons of FeNi_2_Se_4_ are transferred to the C sphere because of the reducing effect of the aldehyde group in glucose. In addition, in the case of the C 1s spectrum of C@FeNi_2_Se_4_, the peaks at the binding energies of 284.8 and 285.7 eV could be assigned to the C─C and C═O bonds, respectively, arising from the C spheres generated from glucose conversion (Figure S3b). The XPS results highlight the synergistic regulation of the electronic structure in C@FeNi_2_Se_4_, where the construction of a core‐shell structure in C@FeNi_2_Se_4_ primarily regulates FeNi_2_Se_4_, facilitating multistage charge redistribution.

### 2.2 Electrocatalytic OER Performance in Alkaline Simulated Seawater

To investigate the OER performance of C@FeNi_2_Se_4_ and control samples (FeNi_2_Se_4_), typical cyclic voltammetry (CV) curves were carried out in 1.0 M KOH + 0.5 M NaCl solution at a scan rate of 100 mV s^−1^ with IR compensation. The evolution of CV curves of C@FeNi_2_Se_4_ for OER during 50 cycles show gradually increased current density and dynamic redox peaks ascribed to the transformation of Ni^2+^ to Ni^3+^, implying the reconstruction of C@FeNi_2_Se_4_ under the applied positive potentials (Figure 2a). The onset potential of Ni oxidation peak gradually decreases, accompanying the cathodic peak shifts negatively (△E = 71 mV), which suggest that the active species (Ni^3+^) form at a lower potential. As for pure FeNi_2_Se_4_, both the cathodic peak shift (△E = 42 mV) and the onset potential of Ni oxidation peak shift become smaller than those of C@FeNi_2_Se_4_ under the same conditions (Figure 2b). Meanwhile, the constant current test curve shows that C@FeNi_2_Se_4_ exhibits a rapid current drop process and eventually stabilizes at 1.51 V, <1.57 V of FeNi_2_Se_4_ (Figure 2c). The above analysis of CV curves suggests that the introduction of C spheres is more favorable of surface reconstruction and promotes the improvement of the catalytic activity of OER. The elemental content in the solution after reconstruction was detected by ICP, indicating that the dissolution of Se led to the reconstruction process of the catalyst. In addition, the amount of Se, Ni, and Fe elements dissolved from C@FeNi_2_Se_4_ was less than that from FeNi_2_Se_4_, suggesting that the introduction of C inhibited the dissolution of these elements (Figure 2d).

The OER performance of the reconstructed FeNi_2_Se_4_ and C@FeNi_2_Se_4_ (Re‐FeNi_2_Se_4_ and Re‐C@FeNi_2_Se_4_) was evaluated in 1.0 M KOH + 0.5 M NaCl solution using a three‐electrode system. To optimize the Re‐C@FeNi_2_Se_4_, the OER performance of Re‐C@FeNi_2_Se_4_ with various amounts of glucose were systematically investigated (Figure S4). As shown in Figure 2e, Re‐C_0.9_@FeNi_2_Se_4_ exhibits the best catalytic performance, and delivers a low overpotential of 223 and 310 mV to achieve current densities of 10 and 100 mA cm^−2^, respectively, which are significantly lower than those (253 and 390 mV) of FeNi_2_Se_4_. To further elucidate the impact of C spheres on OER activity, the Tafel slope as a descriptor of reaction kinetics was analyzed (Figure 2e). Re‐C@FeNi_2_Se_4_ exhibits a Tafel slope of 39.4 mV dec^−1^, which is significantly lower than that of FeNi_2_Se_4_ (65.7 mV dec^−1^), demonstrating the faster catalytic kinetics for Re‐C@FeNi_2_Se_4_. Additionally, the electrochemically active surface area (ECSA), estimated from the double‐layer capacitance (C_dl_) extracted from CV curves in the non‐faradic region (Figure S5), further confirms the enhancement of catalytic performance. Re‐C@FeNi_2_Se_4_ exhibits a significantly larger C_dl_ value of 0.32 mF cm^−2^ compared to Re‐FeNi_2_Se_4_ (0.27 mF cm^−2^), confirming that the C spheres effectively increase the active surface area and facilitate mass transport. The corrosion polarization plots were further evaluated to analyst the anti‐corrosion of samples. Compared with Re‐FeNi_2_Se_4_ (E_corr_ = 0.22 V, j_corr_ = 0.012 mA cm^−2^), Re‐C@FeNi_2_Se_4_ exhibits higher corrosion potential (E_corr_ = 0.26 V) and a lower corrosion current density (j_corr_ = 0.007 mA cm^−2^). It indicates that Re‐C@FeNi_2_Se_4_ can effectively serve two purposes simultaneously: enhancing chloride corrosion resistance and improving catalytic activity (Figure 2g). The durability of Re‐C@FeNi_2_Se_4_ was further evaluated via chronopotentiometry at current densities of 200 and 400 mA cm^−2^ over a continuous 150 h operation, aiming to assess its practical feasibility for industrial applications (Figure 2h). No fluctuations in potential were observed during the test in 1 M KOH + 0.5 M NaCl, indicating the remarkable long‐term durability of Re‐C@FeNi_2_Se_4_. The catalytic performance strikingly surpasses most of the currently reported advanced catalysts (Table S1).

### 2.3 Reconstruction of C@FeNi_2_Se_4_ during the OER Process

**FIGURE 3:**
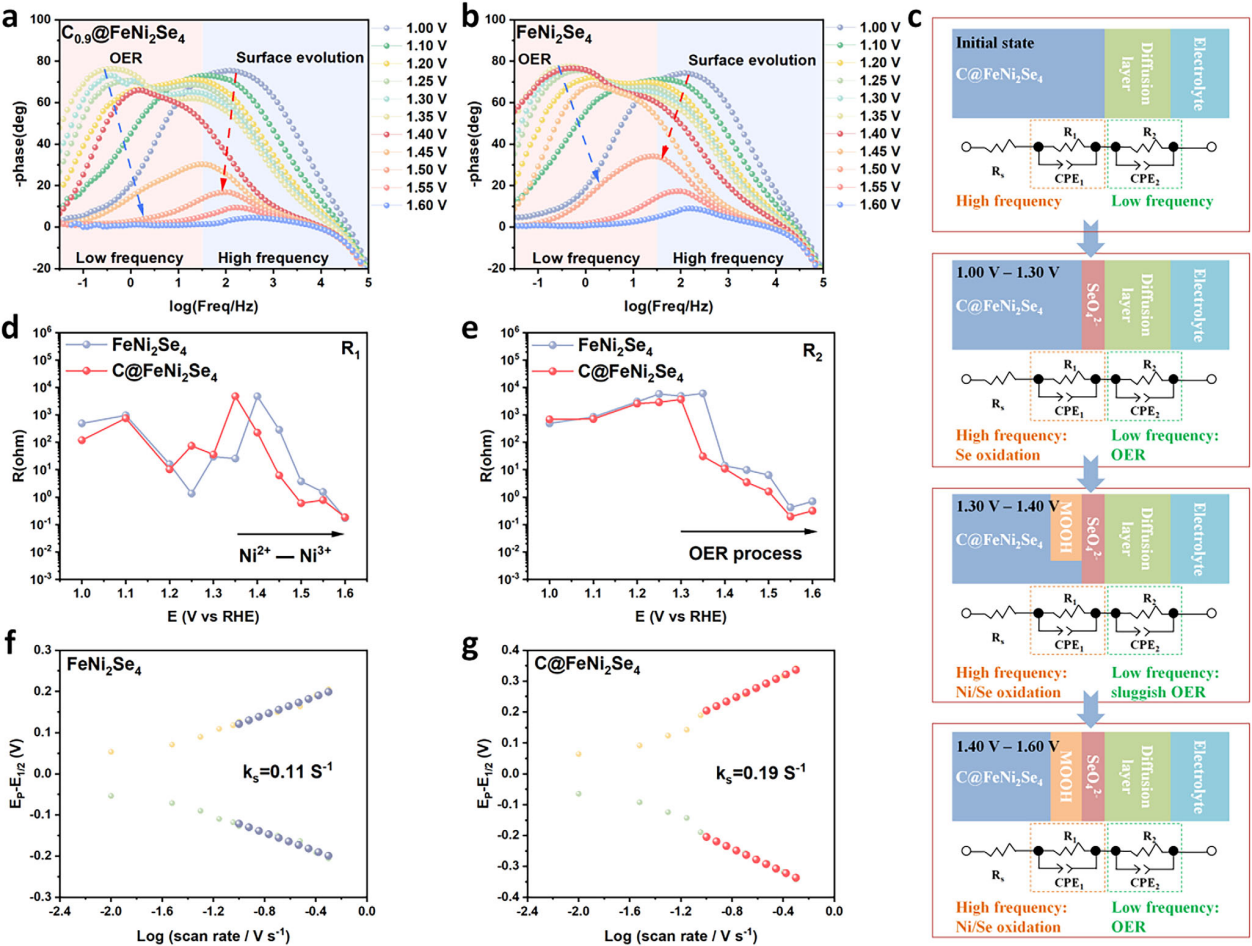
Bode plots of (a) FeNi_2_Se_4_ and (b) C@FeNi_2_Se_4_ for OER at different potentials in 1 M KOH + 0.5 M NaCl. (c) Equivalent circuit models for OER of C@FeNi_2_Se_4_ at different potentials. (d,e) Correlation of Equivalent resistances (R_1_ and R_2_) and potential of FeNi_2_Se_4_ and C@FeNi_2_Se_4_ for OER at different potentials in 1 M KOH + 0.5 M NaCl, where R_1_ and R_2_ are the resistances at high and low frequencies, respectively. Fitted k_s_ of (f) FeNi_2_Se_4_ and (g) C@FeNi_2_Se_4_ in 1 M KOH + 0.5 M NaCl.

Generally, the metal species on the surface of catalysts undergo in‐situ reconstruction during the OER process. The operando Bode plots indicate that the charge transfer signals at different characteristic frequencies can be divided into two areas. Specially, the electron transfer response in the inner layer of the catalyst occurs at the high‐frequency range of 10^1.5^–10^5^ Hz, whereas the charge transfer response at the catalyst/electrolyte interface is observed at the low‐frequency range of 10^−2^–10^1.5^ Hz [44]. Compared to FeNi_2_Se_4_ (Figure 3a), C@FeNi_2_Se_4_ exhibits lower peaks in both high and low frequency ranges, with the peaks decreasing more rapidly as the potential increases (Figure 3b). This suggests a faster oxidation of intermediates and deprotonation of *OOH in C@FeNi_2_Se_4_, revealing significantly enhanced OER activity. The Nyquist plots in Figure S6 are fitted based on a hypothetical equivalent circuit model composed of three parts (Figure 3c, Tables S2 and S3). In detail, the R_s_ stands for the electrolyte resistance. The first parallel circuit involves constant phase element (CPE_1_) and resistance (R_1_) related to the electron transfer from the inner layer of catalyst to the reaction interface, and R_1_ represents the surface reconstruction of the catalyst during OER (Figure 3d). The second parallel circuit includes a constant phase element (CPE_2_) and resistance (R_2_) associated with the charge transfer of interface reaction (Figure 3e).

Analogous trends were observed for C@FeNi_2_Se_4_ and FeNi_2_Se_4_ in terms of their corresponding R_1_ and R_2_ in the 1.00–1.60 V range, indicating their OER performance in this potential window. At the range from 1.00–1.10 V, the C@FeNi_2_Se_4_ showed the smallest equivalent resistance R_1_ (high‐frequency region), which was attributed to rapid electron transfer. After reaching 1.35 V, R_1_ decreased sharply, indicating the onset of catalyzed electrooxidation of M (Ni and Fe) to MOOH (NiFeOOH). And, the R_1_ value of C@FeNi_2_Se_4_ drops sharply at 1.35 V earlier than that of FeNi_2_Se_4_ (1.40 V), manifesting that C@FeNi_2_Se_4_ is more prone to reconstruction than FeNi_2_Se_4_. At the low potential range of 1.30–1.40 V, the R_2_ values (low‐frequency region) of C@FeNi_2_Se_4_ drop sharply but remain large, manifesting that the catalysts occur rapid structural changes accompanied by a sluggish OER process. However, the rapid decrease in R_2_ value of C@FeNi_2_Se_4_ occurred at 1.30 V, which is lower than that of FeNi_2_Se_4_ (1.35 V). The R_2_ value of C@FeNi_2_Se_4_ approaches the low resistance when the potential exceeds 1.40 V indicates the occurrence of OER process. More importantly, within the potential range of 1.40–1.60 V, C@FeNi_2_Se_4_ exhibits a lower resistance than FeNi_2_Se_4_, indicating the superior OER kinetics and intrinsic activity of C@FeNi_2_Se_4_. The introduction of C spheres enhances the utilization rate of *OH, endowing C@FeNi_2_Se_4_ with improved charge transfer and deprotonation capabilities. Additionally, the reconstructing interface of C@FeNi_2_Se_4_ further facilitates OER activity. Under alkaline OER conditions, the redox process of active sites is typically accompanied by the transfer of hydroxide ions (OH^−^). Using the Laviron method, the redox constants (k_s_) were determined. As shown in Figure 3f,g, the k_s_ of C@FeNi_2_Se_4_ (0.19 s^−1^) is higher than that of FeNi_2_Se_4_ (0.11 s^−1^), suggesting faster diffusion of OH^−^ from the electrolyte to the C@FeNi_2_Se_4_ electrode.

### 2.4 OER Mechanism Investigation

**FIGURE 4:**
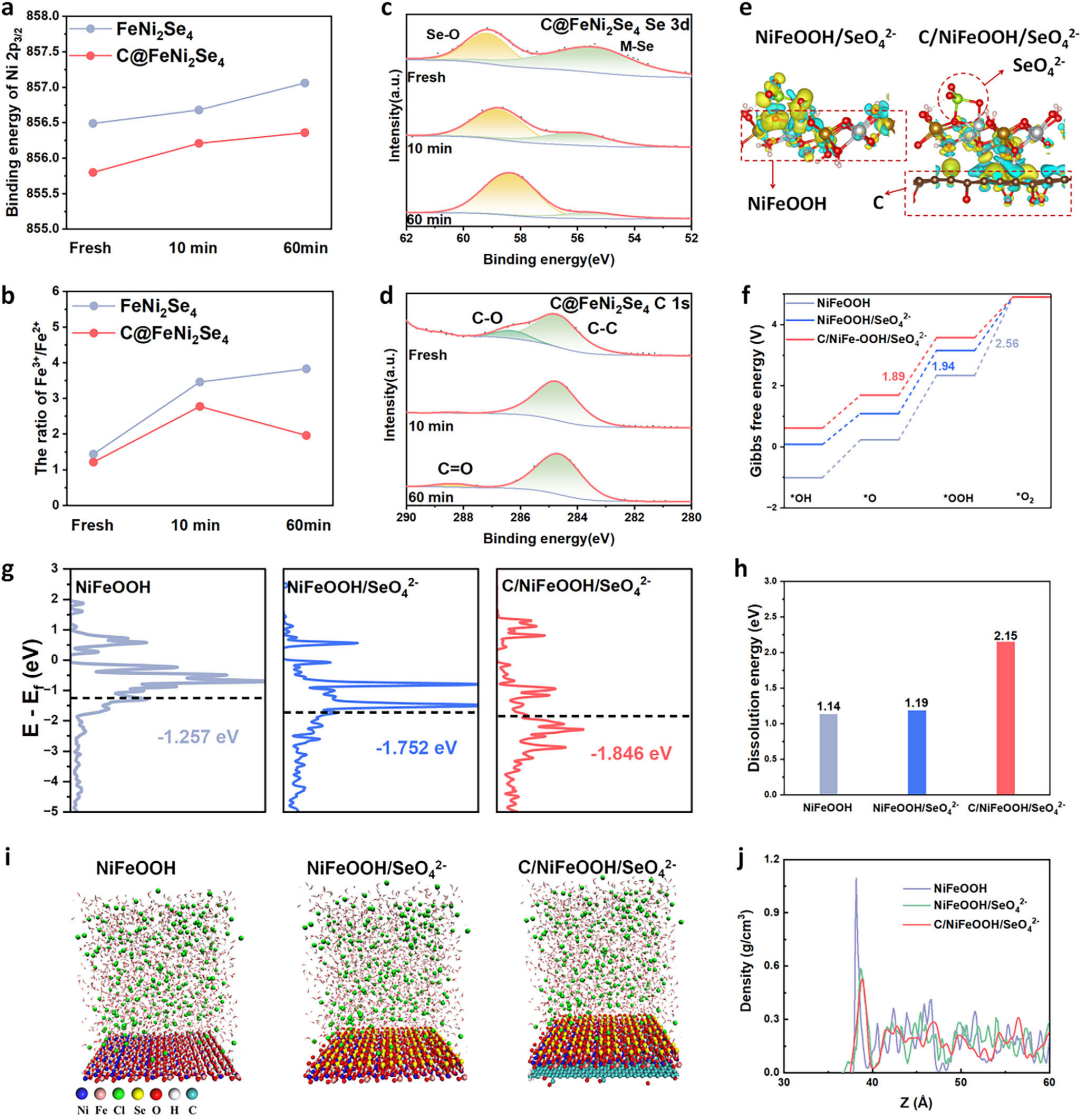
(a) The binding energy of Ni 2p_3/2_ for FeNi_2_Se_4_ and C@FeNi_2_Se_4_ after 10 and 60 min of OER at 100 mA cm^−2^. (b) The ratio of Fe^3+^/Fe^2+^ for FeNi_2_Se_4_ and C@FeNi_2_Se_4_ after 10 and 60 min of OER at 100 mA cm^−2^. XPS spectra of Se 3d (c) and C1s (d) for C@FeNi_2_Se_4_ after 10 and 60 min of OER at 100 mA cm^−2^. (e) Charge density difference of NiFeOOH/SeO_4_^2−^ and C/NiFeOOH/SeO_4_^2−^ (yellow indicates charge accumulation, blue indicates charge depletion). (f) OER pathways and energy barriers of NiFeOOH, NiFeOOH/SeO_4_^2−^, and C/NiFeOOH/SeO_4_^2−^. DOS (g) and dissolution energy (h) of NiFeOOH, NiFeOOH/SeO_4_^2−^, and C/NiFeOOH/SeO_4_^2−^. Snapshots of classical molecular dynamics simulations (i) and the radial distribution density of Cl^−^ (j) versus the distance above NiFeOOH, NiFeOOH/SeO_4_^2−^, and C/NiFeOOH/SeO_4_^2−^ under 1 M KOH + 0.5 M NaCl.

To gain deeper insight into the surface dynamic reconstruction of C@FeNi_2_Se_4_ during the OER, its structural evolution was examined in a three‐electrode system. The XRD results of the catalyst before and after reconstruction exhibit that Re‐C@FeNi_2_Se_4_ shows a lower crystallinity and a wider half‐peak width compared with C@FeNi_2_Se_4_, indicating that the reconstruction phenomenon occurs on the surface of the catalyst (Figure S7). In situ Raman is employed to investigate the surface structure evolution of catalysts in 1 M KOH + 0.5 M NaCl solution under oxidation voltage. As shown in Figure S8, a weak Se‐Se vibrational mode (∼332 cm^−1^) can be observed at open‐circuit potential before gradually diminishing with increasing potential, indicating that Se species dissolve during the OER process. Meanwhile, the occurrence of notable Raman peaks at 455 and 531 cm^−1^, corresponding to the E_g_ Ni‐O bending vibration mode and the A_1g_ Ni‐O stretching vibration, demonstrated the generation of the NiOOH phase. An additional new band occurred at 844 cm^−1^, with an increased potential ranging from 1.521 to 1.621 V, which is related to SeO_4_^2−^. The in situ Raman characterization results indicate that the C@FeNi_2_Se_4_ catalyst is in situ transformed into C/NiFeOOH/SeO_4_^2−^ at the oxidation potential, accompanied by the leaching of Se. Furthermore, evidence for this reconstruction process was obtained by XPS analysis of FeNi_2_Se_4_ and C@FeNi_2_Se_4_ after 10 and 60 min. In order to further analyze the process of surface reconstruction phenomena, we conducted a further analysis of the valence states of Ni and Fe. Ni 2p3/2 peaks in FeNi_2_Se_4_ and C@FeNi_2_Se_4_ were shift toward the high energy direction with the increased potential, providing evidence for the formation of high valence active species (Figure 4a, Figure S9a,b). However, the valence state of Ni of C@FeNi_2_Se_4_ is lower than that of FeNi_2_Se_4_, indicating the introduction of C stabilized the valence state of NiOOH during the reconstruction process. Similarly, a continuous increase in the Fe^3^^+^/Fe^2^^+^ ratio was observed for both FeNi_2_Se_4_ and C@FeNi_2_Se_4_, indicating the Fe was oxidized to FeOOH during the reconstruction process. Subsequently, the proportions in FeNi_2_Se_4_ gradually increased. Note that the ratio of Fe^3+^/Fe^2+^ in C@FeNi_2_Se_4_ gradually decreases (Figure 4b, Figure S9c,d). The Ni 2p spectrum of C@FeNi_2_Se_4_ shows peaks at 855.9 and 852.6 eV, corresponding the Ni 2p_3/2_ and Ni‐Se bonds. Notably, in C@FeNi_2_Se_4_, the Ni─Se peaks disappear, indicating complete surface reconstructions. Concurrently, Fe─Se bond observed at 706.7 eV in Fe 2p spectrum also disappears during the oxidation process. These observations confirm that the OER process triggers the dissolution of Se atoms, leading to comprehensive surface reconstruction of the catalyst. Furthermore, the Se 3d spectrum of C@FeNi_2_Se_4_ shows peaks at 55.4 and 59.2 eV, corresponding to M─Se and Se─O. In Se 3d spectrum of FeNi_2_Se_4_, the Se─O peak gradually disappears, indicating the gradual transformation of FeNi_2_Se_4_ into NiFeOOH (Figure S10). Notably, the M‐Se peaks gradually weakened, and the Se─O peak gradually strengthened, indicating that Se dissolved to form SeO_4_^2−^ species during the reconstruction process and then adsorbed onto the surface of the catalyst (Figure 4c). In addition, the C 1s spectrum in C@FeNi_2_Se_4_ revealed the conversion of C─O bonds to C═O bonds during the OER process (Figure 4d). Therefore, C@FeNi_2_Se_4_ undergoes an in‐situ transformation into a sandwich‐like architecture, comprising a carbon core, a reconstructed NiFeOOH layer, and an outermost surface layer of adsorbed selenate anions.

Density functional theory (DFT) calculations were performed to investigate the electronic structures of the catalysts and their OER mechanism. Figure 4e presents the charge density difference of NiFeOOH/SeO_4_^2−^ and C/NiFeOOH/SeO_4_^2−^. The charge redistribution between NiFeOOH and SeO_4_^2−^ indicates strong electronic interactions at their interface. The excess electrons of SeO_4_^2−^ tend to transfer to NiFeOOH. Similarly, charge redistribution in C/NiFeOOH/SeO_4_^2−^ suggests electron donation from carbon to NiFeOOH, indicating that C modifies the electronic structure. This redistribution further leads to a reduction in the valence states of Ni and Fe in the reconstructed C@FeNi_2_Se_4_ compared to the reconstructed FeNi_2_Se_4_. Different from the AEM pathway, the LOM mechanism follows non‐concerted proton‐electron transfer kinetics, arising from a mismatch between electron transfer involving lattice oxygen and metal and OH^−^ adsorption/reaction at the catalyst/electrolyte interface, which exhibits a strong pH dependence. As illustrated in Figure S11, though the OER activity of NiFeOOH/SeO_4_^2−^ and C/NiFeOOH/SeO_4_^2−^ slightly increased with increasing pH, the proton reaction order on the RHE scale was relatively low. It indicates that the OER on C/NiFeOOH/SeO_4_^2−^ primarily undergoes an AEM pathway [45, 46]. To further elucidate the OER process, the energy barriers for key intermediated transformations were analyzed by the adsorbate evolution mechanism (AEM). Figure S12 illustrates the adsorption model and a schematic representation of the potential OER pathway on NiFeOOH, NiFeOOH/SeO_4_^2−^, and C/NiFeOOH/SeO_4_^2−^. To determine the active site, the Gibbs free energy of *OH was calculated on both Ni and Fe centers within the catalyst model. The *OH adsorption energy on the Ni sites of NiFeOOH, NiFeOOH/SeO_4_^2−^, and C/NiFeOOH/SeO_4_^2−^ is significantly stronger compared to that on the Fe site, confirming that Ni is the principal active center (Figure S13). In addition, the ICP result of C/NiFeOOH/SeO_4_^2−^ indicates that the ratio of Ni to Fe content in the catalyst is 2:1 (Figure S14). Ni serves as the main active site, and Fe optimizes the electronic structure of the Ni center to promote the generation of high‐valent Ni active species, while stabilizing the *O intermediate in the process of generating *OOH [47]. Figure 4f displays the calculated Gibbs free energy difference (△G) for each step. NiFeOOH exhibits a more negative △G for *OH, *O, and *OOH adsorption, indicating a stronger adsorption affinity toward oxygen‐containing species and enhanced oxophilicity. Therefore, the process of *OOH deprotonation to release O_2_ (*OOH→O_2_) is the rate‐determining step in the OER process and shows the highest energy barrier (△G = 2.56 eV). In contrast, NiFeOOH/SeO_4_^2−^ and C/NiFeOOH/SeO_4_^2−^ exhibit more positive △G for *OH, *O, and *OOH adsorption compared to NiFeOOH, indicating that the introduction of SeO_4_^2−^ and C moderates the adsorption strength of NiFeOOH on *OH, *O, and *OOH and weakens the oxygenicity of NiFeOOH. Notably, the OER rate‐determining step of NiFeOOH/SeO_4_^2−^ and C/NiFeOOH/SeO_4_^2−^ is transformed into the process where *O intermediate reacts with OH^−^ to form *OOH (*O→*OOH) due to the reduction of oxygen affinity. The corresponding △G are 1.94 and 1.89 eV, respectively, indicating that the introduction of SeO_4_^2−^ and C optimizes the reaction process of NiFeOOH and enhances catalytic activity during the OER. According to the d‐band centers theory, the electronic state at the d‐band center determines the adsorption capacity of the metal for the reactants. When the d‐band center is relatively high, it indicates that the electronic energy level of the d orbitals on the metal surface is closer to the Fermi level, thereby resulting in a stronger affinity for oxygen‐containing reaction intermediates (*OH, *O, *OOH) on the electrocatalyst surface [48, 49]. Moreover, the density of state (DOS) indicates that the d band center of Ni sites in NiFeOOH/SeO_4_^2−^ and C/NiFeOOH/SeO_4_^2−^ (−1.257 eV) is further away from the Fermi level than that of NiFeOOH, which favors a weaker bonding with the OER intermediates (Figure 4g). Notably, the energy barrier of the rate‐determining step and d bond center of NiFeOOH/SeO_4_^2−^ and C/NiFeOOH/SeO_4_^2−^ are close, indicating that the electronic interaction between SeO_4_^2−^ and NiFeOOH plays a key role in reducing the surface oxyphilicity of the NiFeOOH and optimizing the reaction energy barrier. Furthermore, the dissolution energy of the above models was then determined to reveal the inhibitory effect of C on the metal dissolution of NiFeOOH, thereby enhancing its structural improve stability (Figure 4h). As expected, the dissolution energies of mental increased from 1.14 to 1.19 and 2.15 eV for NiFeOOH/SeO_4_^2−^ and C/NiFeOOH/SeO_4_^2−^, respectively. The increase in metal dissolution energy of C/NiFeOOH/SeO_4_^2−^ compared to NiFeOOH and NiFeOOH/SeO_4_^2−^ suggests a synergistic stabilization effect between NiFeOOH and C. C can further enhance the binding strength of the NiFeOOH, which can be attributed to its electron‐donating effect. Furthermore, the classical Molecular Dynamics (MD) simulations were carried out to deeply study the role of SeO_4_^2−^ in the improvement of anode corrosion resistance and stability. As shown in Figure 4i, Cl^−^ ions tend to accumulate near the NiFeOOH surface while staying away from the NiFeOOH/SeO_4_^2^ and C/NiFeOOH/SeO_4_^2−^ due to the electrostatic repulsion. The radial distribution density versus the distance illustrates the concentration of Cl^−^ increases rapidly on the surface of NiFeOOH, reaching a peak at 38.17 Å. Compared to the NiFeOOH, the concentration of Cl^−^ on the NiFeOOH/SeO_4_^2^ and C/NiFeOOH/SeO_4_^2−^ decreases significantly and reaches its peak at 38.65 and 38.83 Å (Figure 4j), respectively. It is clear that the presence of SeO_4_^2−^ makes Cl^−^ difficult to approach the surface, which as Cl^−^ repelling armor to protect active sites during the OER process. Therefore, both SeO_4_^2−^ and C in C/NiFeOOH/SeO_4_^2−^ serve as the primary active component that contributes to the enhanced activity and stability of OER, which is consistent with the experimental observations.

### 2.5 Electrocatalytic OER Performance Under Industrial Conditions

**FIGURE 5:**
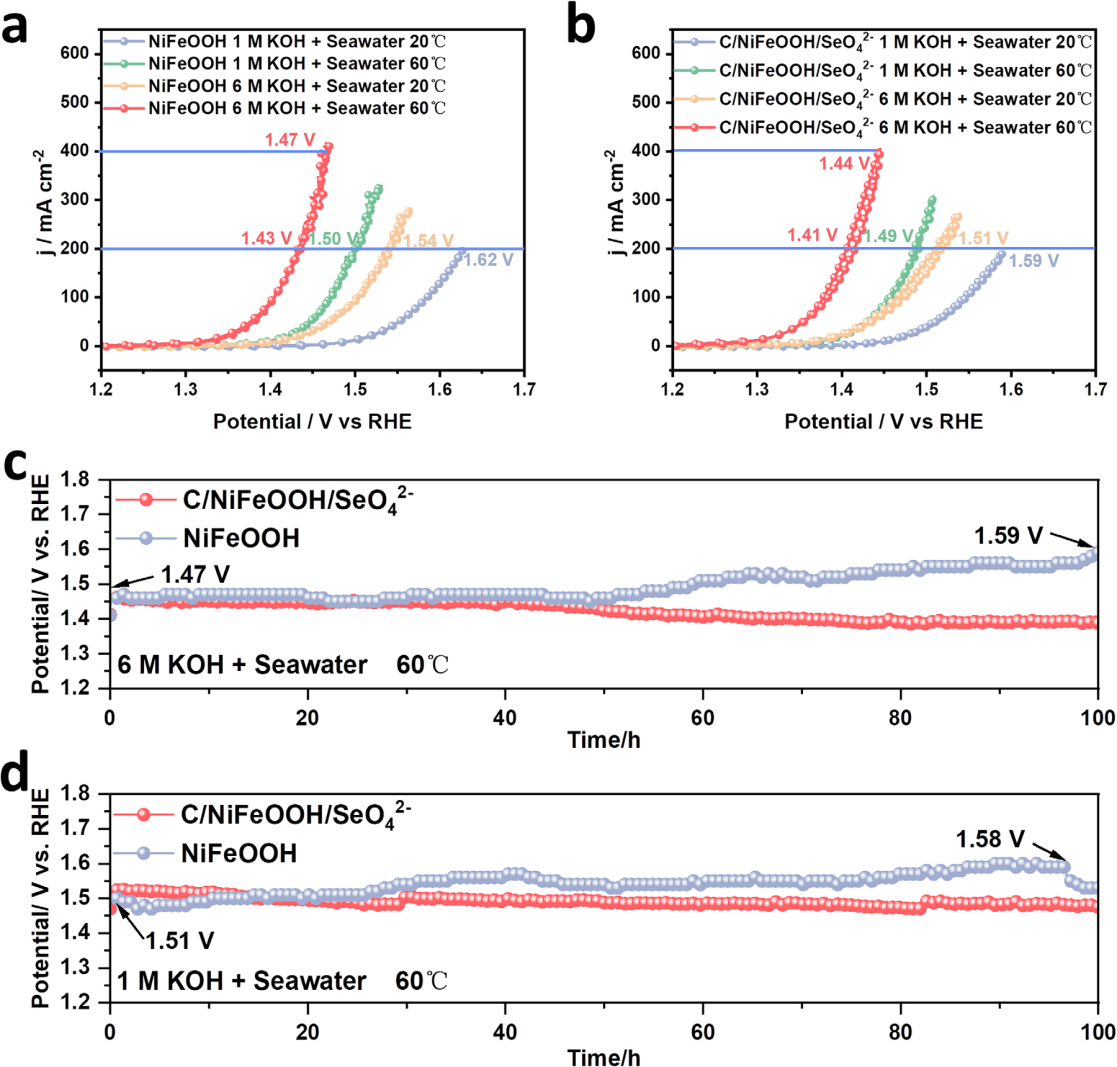
The CV curves of (a) NiFeOOH and (b) C/NiFeOOH/SeO_4_^2−^, in 1 M KOH + Seawater, 6 M KOH + Seawater at 20°C and 60°C, respectively. Durability tests of NiFeOOH and C/NiFeOOH/SeO_4_^2−^ at a constant current density of 200 mA cm^−2^ in (c) 6 M KOH + Seawater and (d) 1 M KOH + Seawater, respectively.

To evaluate the applicability of C/NiFeOOH/SeO_4_^2−^ for splitting seawater in industrial applications, we can assume that the C/NiFeOOH/SeO_4_^2−^ electrode possesses ultra‐long operation life for OER. In experiments, the actual alkaline seawater electrolyte (1 M KOH + seawater and 6 M KOH + seawater) was first adapted for accelerated evaluation of anode lifetime with a highly stable Pt foil cathode under high temperature. The results in Figure 5a demonstrated that NiFeOOH required potentials of 1.62 and 1.50 V to achieve the current densities of 200 mA cm^−2^ under 20°C and 60°C in 1 M KOH + seawater, respectively. And in 6 M KOH + seawater electrolyte, the potential of NiFeOOH is 1.54 and 1.43 V at the current density of 200 mA cm^−2^ under 20°C and 60°C, respectively. Moreover, in 1 M KOH + seawater, C/NiFeOOH/SeO_4_^2−^ exhibited much lower potentials (1.59 and 1.49 V) for current density of 200 mA cm^−2^ at 20°C and 60°C, respectively. Furthermore, C/NiFeOOH/SeO_4_^2−^ requires potential of only 1.51 and 1.41 V to achieve the current density of 200 mA cm^−2^ at 20°C and 60°C, respectively (Figure 5b), demonstrating remarkable catalytic activity for industrial applications. Notably, durability is an important performance metric for catalysts in industrial production. First, 6 M KOH + seawater was used as the electrolyte for accelerated experiments. In these experiments, the potential of the NiFeOOH sample increased by 120 mV when it works continuously for 100 h at a current density of 200 mA cm^−2^ under 60°C, while the C/NiFeOOH/SeO_4_^2−^ endured for more than 100 h without performance decline (Figure 5c). Figure 5d shows that C/NiFeOOH/SeO_4_^2−^ endured for 100 h without obvious loss of OER performance in 1 M KOH + Seawater under 60°C, while the potential of the NiFeOOH increased by 60 mV after 100 h of stable operation. As shown in Figure S15, the metal dissolution amount for C/NiFeOOH/SeO_4_^2−^ was much lower than that for NiFeOOH, further proving the protective effect of internal and external synergy. Meanwhile, to mitigate environmental risks and reclaim valuable selenium resources for electronics and photovoltaics, SeO_4_^2−^ in the electrolyte could be recovered through physical (nanofiltration or Reverse osmosis) or chemical methods (Ion exchange adsorption or Oxidation/reduction) during industrial applications [50]. Clearly, the C/NiFeOOH/SeO_4_^2−^ achieves a leap‐forward development in the performance of seawater electrolysis, showing great promise for industry application.

## 3 Conclusions

In summary, an internal and external synergy was proposed to achieve efficient and stable seawater electrolysis through controlling reconstruction. C@FeNi_2_Se_4_ pre‐catalyst with a core‐shell structure was successfully prepared to enhance the performance of oxygen evolution reaction (OER). During the OER operation, the pre‐catalyst undergoes a controlled surface reconstruction into C/NiFeOOH/SeO_4_^2−^, significantly enhancing corrosion resistance, catalytic activity, and structural stability. As a result, the C/NiFeOOH/SeO_4_^2−^ anode achieves exceptionally low overpotentials of 223 and 310 mV at 10 and 100 mA cm^−2^, respectively, in simulated seawater (1 M KOH + 0.5 M NaCl). Moreover, it demonstrates outstanding durability, maintaining stable operation for 150 h at 400 mA cm^−^^2^. Note that the C/NiFeOOH/SeO_4_^2−^ sustains stable electrolysis of seawater over 100 h in alkaline seawater (6 M KOH + seawater) at industrial conditions (60°C, 200 mA cm^−2^). Combined experimental and density functional theory (DFT) calculations reveal that the C@FeNi_2_Se_4_ core‐shell structure accelerates surface reconstruction to form C/NiFeOOH/SeO_4_^2−^. The electrons on SeO_4_^2−^ and C are transferred to NiFeOOH, keeping NiFeOOH in a lower valence state and achieving electron redistribution. The adsorbed SeO_4_^2−^ anion layer on the NiFeOOH surface effectively repels chloride ions, thereby enhancing corrosion resistance. Furthermore, SeO_4_^2−^ optimizes the d‐band center, weakens the hydrogen‐oxygen property of the NiFeOOH surface, reduces the adsorption of oxygen‐containing intermediates, and changes the rate‐determining step from *OOH‐*O_2_ to *O‐*OOH process. A strong electronic interaction occurs between the inner C and NiFeOOH, increasing the dissolution potential of the metal sites and further promoting the stability of OER. These findings highlight the potential of utilizing transition metal selenides reconstruction to optimize OER performance, paving the way for the practical application of seawater electrolysis. The self‐reconstructed C/NiFeOOH/SeO_4_^2−^, leveraging internal and external synergistic effects, presents a novel strategy for designing highly efficient and durable electrocatalysts for seawater splitting.

## Conflicts of Interest

The authors declare no conflict of interest.

## Supporting information




**Supporting File**: advs74112‐sup‐0001‐SuppMat.docx

## Data Availability

The data that support the findings of this study are available from the corresponding author upon reasonable request.

## References

[1] M. He , Y. Sun , and B. Han , “Green Carbon Science: Efficient Carbon Resource Processing, Utilization, and Recycling toward Carbon Neutrality,” Angewandte Chemie International Edition 61 (2022): 202112835, 10.1002/anie.202112835. 34919305

[2] T.‐Z. Ang , M. Salem , M. Kamarol , H. S. Das , M. A. Nazari , and N. Prabaharan , “A Comprehensive Study of Renewable Energy Sources: Classifications, Challenges and Suggestions,” Energy Strategy Reviews 43 (2022): 100939, 10.1016/j.esr.2022.100939.

[3] P. Li , Y. Yao , W. Ouyang , Z. Liu , H. Yin , and D. Wang , “A Stable Oxygen Evolution Splitting Electrocatalysts High Entropy Alloy FeCoNiMnMo in Simulated Seawater,” Journal of Materials Science & Technology 138 (2023): 29–35, 10.1016/j.jmst.2022.08.012.

[4] L. Hu , X. Tan , X. Yang , and K. Zhang , “Electrolysis of Direct Seawater: Challenges, Strategies, and Future Prospects †,” Chinese Journal of Chemistry 41 (2023): 3484–3492, 10.1002/cjoc.202300324.

[5] P. Guo , D. Liu , and R. Wu , “Recent Progress in Design Strategy of Anode for Seawater Electrolysis,” Small Structures 4 (2023): 2300192, 10.1002/sstr.202300192.

[6] P. Li , S. Zhao , Y. Huang , et al., “Corrosion Resistant Multilayered Electrode Comprising Ni_3_N Nanoarray Overcoated With NiFe‐Phytate Complex for Boosted Oxygen Evolution in Seawater Electrolysis,” Advanced Energy Materials 14 (2023): 2303360, 10.1002/aenm.202303360.

[7] S. Dresp , F. Dionigi , M. Klingenhof , and P. Strasser , “Direct Electrolytic Splitting of Seawater: Opportunities and Challenges,” ACS Energy Letters 4 (2019): 933–942.

[8] Y. Song , M. Sun , S. Zhang , et al., “Alleviating the Work Function of Vein‐Like Co_X_P by Cr Doping for Enhanced Seawater Electrolysis,” Advanced Functional Materials 33 (2023): 2214081, 10.1002/adfm.202214081.

[9] H. Jin , J. Xu , H. Liu , et al., “Emerging Materials and Technologies for Electrocatalytic Seawater Splitting,” Science Advances 9 (2023): adi7755, 10.1126/sciadv.adi7755. PMC10584342 37851797

[10] T. Liu , Z. Zhao , W. Tang , et al., “In‐Situ Direct Seawater Electrolysis Using Floating Platform In Ocean With Uncontrollable Wave Motion,” Nature Communications 15 (2024): 5305, 10.1038/s41467-024-49639-6. PMC11192878 38906873

[11] J. Liang , Z. Li , X. He , et al., “Electrocatalytic Seawater Splitting: Nice Designs, Advanced Strategies, Challenges and Perspectives,” Materials Today 69 (2023): 193–235, 10.1016/j.mattod.2023.08.024.

[12] W. Liu , J. Yu , T. Li , et al., “Self‐Protecting CoFeAl‐Layered Double Hydroxides Enable Stable And Efficient Brine Oxidation at 2 A cm^−2^ ,” Nature Communications 15 (2024): 4712, 10.1038/s41467-024-49195-z. PMC11148009 38830888

[13] X. Sun , W. Shen , H. Liu , et al., “Corrosion‐Resistant NiFe Anode Toward Kilowatt‐Scale Alkaline Seawater Electrolysis,” Nature Communications 15 (2024): 10351, 10.1038/s41467-024-54754-5. PMC11605038 39609468

[14] W. Liu , X. Ding , J. Cheng , et al., “Inhibiting Dissolution of Active Sites in 80 °C Alkaline Water Electrolysis by Oxyanion Engineering,” Angewandte Chemie International Edition 63 (2024): 202406082, 10.1002/anie.202406082. 38807303

[15] H. Yu , J. Wan , M. Goodsite , and H. Jin , “Advancing Direct Seawater Electrocatalysis for Green and Affordable Hydrogen,” One Earth 6 (2023): 267–277, 10.1016/j.oneear.2023.02.003.

[16] F. Wang , X. Zhao , Y. Li , et al., “Constructing Reconstruction‐Inhibited Nickel Selenide Electrocatalysts Via Incorporating Ag Single Atom for Durable and Efficient Water Oxidation,” Applied Catalysis B: Environment and Energy 348 (2024): 123830, 10.1016/j.apcatb.2024.123830.

[17] S. Zhang , Y. Wang , S. Li , et al., “Concerning the Stability of Seawater Electrolysis: A Corrosion Mechanism Study of Halide on Ni‐Based Anode,” Nature Communications 14 (2023): 4822, 10.1038/s41467-023-40563-9. PMC10415325 37563114

[18] D. Liu , P. Guo , H. Pan , and R. Wu , “Emerging High‐Entropy Compounds for Electrochemical Energy Storage and Conversion,” Progress in Materials Science 145 (2024): 101300, 10.1016/j.pmatsci.2024.101300.

[19] W. Xu , Z. Wang , P. Liu , et al., “Ag Nanoparticle‐Induced Surface Chloride Immobilization Strategy Enables Stable Seawater Electrolysis,” Advanced Materials 36 (2023): 2306062, 10.1002/adma.202306062. 37907201

[20] L. Wu , F. Zhang , S. Song , et al., “Efficient Alkaline Water/Seawater Hydrogen Evolution by a Nanorod‐Nanoparticle‐Structured Ni‐MoN Catalyst With Fast Water‐Dissociation Kinetics,” Advanced Materials 34 (2022): 2201774, 10.1002/adma.202201774. 35363922

[21] X. Zhang , Q. Hou , S. Cao , et al., “Research Status, Opportunities, and Challenges of Cobalt Phosphate Based Materials as OER Electrocatalysts,” Green Chemistry 25 (2023): 7883–7903, 10.1039/D3GC02416D.

[22] X. Zhang , X. Lin , S. Cao , et al., “Strong Electron Interaction at the Amorphous/Crystalline Interface Enables Advanced Oxygen Evolution Reaction,” ACS Sustainable Chemistry & Engineering 12 (2024): 3175–3184, 10.1021/acssuschemeng.3c07189.

[23] X. Zhang , Z. Wang , S. Cao , et al., “Breaking the Scaling Relationship For High‐Performance Seawater Oxidation Through Lattice Distortion Triggered By Molybdenum,” Journal of Materials Science & Technology 225 (2025): 165–173, 10.1016/j.jmst.2024.10.050.

[24] X. Zhang , H. Zhang , Z. Chen , et al., “Self‐Adapting Oxyanion Armor Achieves Highly Stable and Efficient Seawater Electrolysis at Ampere‐Level Current Densities,” Advanced Functional Materials 35 (2024): 2418940, 10.1002/adfm.202418940.

[25] D. Liu , P. Guo , X. Yan , Y. He , and R. Wu , “Manipulating the Configuration Entropy of Layered Hydroxides Toward Efficient Oxygen Evolution Reaction For Anion Exchange Membrane Electrolyzer,” Materials Today 80 (2024): 101–112, 10.1016/j.mattod.2024.08.008.

[26] K. Huang , J. Xia , Y. Lu , et al., “Self‐Reconstructed Spinel Surface Structure Enabling the Long‐Term Stable Hydrogen Evolution Reaction/Oxygen Evolution Reaction Efficiency of FeCoNiRu High‐Entropy Alloyed Electrocatalyst,” Advanced Science 10 (2023): 2300094, 10.1002/advs.202300094. 36950752 PMC10190517

[27] S. Li , Z. Li , R. Ma , et al., “A Glass‐Ceramic With Accelerated Surface Reconstruction Toward the Efficient Oxygen Evolution Reaction,” Angewandte Chemie International Edition 60 (2020): 3773–3780, 10.1002/anie.202014210. 33174369

[28] D. Liu , P. Guo , Q. Wang , et al., “Local Proton‐Mediated Synthesis of a High‐Entropy Borate Library,” Advanced Materials 37 (2025): 2414067, 10.1002/adma.202414067. 39617977

[29] L. Wu , Z. Guan , D. Guo , L. Yang , X. Chen , and S. Wang , “High‐Efficiency Oxygen Evolution Reaction: Controllable Reconstruction of Surface Interface,” Small 19 (2023): 2304007, 10.1002/smll.202304007. 37551051

[30] Z. Feng , H. Meng , Y. Fu , L. Ren , B. Gao , and W. Liu , “Modulation of Charge Redistribution in Heterogeneous CoSe‐Ni_0.95_Se Coupling With Ti_3_C_2_T_x_ MXene for Hydrazine‐Assisted Water Splitting,” Small 20 (2024): 2403270, 10.1002/smll.202403270. 39444204

[31] R. Zhao , H. Di , X. Hui , et al., “Self‐assembled Ti_3_C_2_MXene and N‐Rich Porous Carbon Hybrids As Superior Anodes For High‐Performance Potassium‐Ion Batteries,” Energy & Environmental Science 13 (2020): 246–257, 10.1039/C9EE03250A.

[32] Y. Sun , H. Lin , C. Wang , Q. Wu , X. Wang , and M. Yang , “Morphology‐Controlled Synthesis of TiO_2_/MoS_2_ Nanocomposites With Enhanced Visible‐Light Photocatalytic Activity,” Inorganic Chemistry Frontiers 5 (2018): 145–152, 10.1039/C7QI00491E.

[33] Z. Li , Y. Yao , S. Sun , et al., “Carbon Oxyanion Self‐Transformation on NiFe Oxalates Enables Long‐Term Ampere‐Level Current Density Seawater Oxidation,” Angewandte Chemie International Edition 63 (2023): 202316522, 10.1002/anie.202316522. 37994225

[34] X. Xu , H. Liao , L. Huang , et al., “Surface Reconstruction And Directed Electron Transport in NiSe_2_/MoSe_2_ Mott‐Schottky Heterojunction Catalysts Promote Urea‐Assisted Water Splitting,” Applied Catalysis B: Environmental 341 (2024): 123312, 10.1016/j.apcatb.2023.123312.

[35] S. Gong , Y. Meng , Z. Jin , H.‐Y. Hsu , M. Du , and F. Liu , “Recent Progress on the Stability of Electrocatalysts Under High Current Densities Toward Industrial Water Splitting,” ACS Catalysis 14 (2024): 14399–14435, 10.1021/acscatal.4c03700.

[36] N. C. S. Selvam , L. Du , B. Y. Xia , P. J. Yoo , and B. You , “Reconstructed Water Oxidation Electrocatalysts: The Impact of Surface Dynamics on Intrinsic Activities,” Advanced Functional Materials 31 (2020): 2008190, 10.1002/adfm.202008190.

[37] J. Feng , X. Wang , and H. Pan , “In‐Situ Reconstruction of Catalyst in Electrocatalysis,” Advanced Materials 36 (2024): 2411688, 10.1002/adma.202411688. 39436113 PMC11635912

[38] F. Zhang , K. Wang , H. Zhang , et al., “Dynamic Reconstruction of Ce‐Doped Fe_2_P/NiCoP Hybrid for Ampere‐Level Oxygen Evolution in Anion Exchange Membrane Water Electrolysis,” Advanced Functional Materials 35 (2025): 2500861.

[39] S. Cai , H. Liu , H. Cheng , et al., “Deeply Reconstructed NiFe Layered Double Hydroxide Nanosheets for an Efficient Oxygen Evolution Reaction,” ACS Applied Nano Materials 6 (2023): 7864–7872.

[40] L. Xiao , X. Bai , J. Han , et al., “Surface Reconstruction and Structural Transformation of Two‐Dimensional Ni‐Fe MOFs for Oxygen Evolution in Seawater Media,” Nano Research 17 (2023): 2429–2437, 10.1007/s12274-023-6088-x.

[41] B. Jia , B. Zhang , Z. Cai , X. Yang , L. Li , and L. Guo , “Construction of Amorphous/Crystalline Heterointerfaces for Enhanced Electrochemical Processes,” eScience 3 (2023): 100112, 10.1016/j.esci.2023.100112.

[42] Y. Chen , Z. Ren , H. Fu , X. Zhang , G. Tian , and H. Fu , “NiSe‐Ni_0.85_Se Heterostructure Nanoflake Arrays on Carbon Paper as Efficient Electrocatalysts for Overall Water Splitting,” Small 14 (2018): 1800763, 10.1002/smll.201800763. 29806149

[43] K. Guo , Z. Zou , J. Du , Y. Zhao , B. Zhou , and C. Xu , “Coupling FeSe_2_ With CoSe: An Effective Strategy to Create Stable and Efficient Electrocatalysts for Water Oxidation,” Chemical Communications 54 (2018): 11140–11143, 10.1039/C8CC06628K. 30225500

[44] Z. Liang , D. Shen , Y. Wei , et al., “Modulating the Electronic Structure of Cobalt‐Vanadium Bimetal Catalysts for High‐Stable Anion Exchange Membrane Water Electrolyzer,” Advanced Materials 36 (2024): 2408634, 10.1002/adma.202408634. 39148167

[45] J. Mu , C. Yu , X. Song , L. Chen , J. Zhao , and J. Qiu , “A Super‐Chlorophobic Yet Weak‐Reconstructed Electrocatalyst by Fluorination Engineering Toward Chlorine Oxidation‐Free and High‐Stability Seawater Electrolysis,” Advanced Functional Materials 35 (2025): 2423965, 10.1002/adfm.202423965.

[46] Z. Li , Y. Yao , S. Sun , et al., “Carbon Oxyanion Self‐Transformation on NiFe Oxalates Enables Long‐Term Ampere‐Level Current Density Seawater Oxidation,” Angewandte Chemie International Edition 63 (2023): 202316522, 10.1002/anie.202316522. 37994225

[47] Y.‐J. Ko , M. H. Han , H. Kim , et al., “Unraveling Ni‐Fe 2D Nanostructure With Enhanced Oxygen Evolution Via In Situ And Operando Spectroscopies,” Chem Catalysis 2 (2022): 2312–2327, 10.1016/j.checat.2022.07.016.

[48] Y. Jiang , P. Qiu , Q. Liu , P. Li , and S. Chen , “Electric‐Double‐Layer Mechanism of Surface Oxophilicity in Regulating the Alkaline Hydrogen Electrocatalytic Kinetics,” Journal of the American Chemical Society 147 (2025): 14122–14130, 10.1021/jacs.4c14511. 40243362

[49] F. Zhang , K. Wang , H. Zhang , et al., “Dynamic Reconstruction of Ce‐Doped Fe_2_P/NiCoP Hybrid for Ampere‐Level Oxygen Evolution in Anion Exchange Membrane Water Electrolysis,” Advanced Functional Materials 35 (2025): 2500861, 10.1002/adfm.202500861.

[50] I. Ali and V. Shrivastava , “Recent advances in Technologies For Removal And Recovery Of Selenium From (Waste)Water: A Systematic Review,” Journal of Environmental Management 294 (2021): 112926, 10.1016/j.jenvman.2021.112926. 34118514

