# Supplementary material for: The Internal‐External Synergy of Self‐Reconstructed C/NiFeOOH/SeO4 2− for Efficient and Stable Seawater Electrolysis
Source: Adv Sci (Weinh). 2026 Jan 27;13(19):e23396. doi: 10.1002/advs.202523396 (PMC13045438; doi:10.1002/advs.202523396)
Supplement: Supplementary file 1 — Supporting File: advs74112‐sup‐0001‐SuppMat.docx [file ADVS-13-e23396-s001.docx]

**Supporting Information**

**The internal-external synergy of self-reconstructed C/NiFeOOH/SeO_4_^2-^** **for efficient and stable seawater electrolysis**

Zhe Sun^a^, Yitong Yin^b^, Siyuan Liu^b^, Bo Liao^a,c^, Bing He^b^*, Zhaojie Wang^b^, Xiaoqing Lu*^b^, Xingheng Zhang^b^*

^a^ Department of Chemical and Petroleum Engineering, University of Calgary, Calgary, Alberta, T2N 1N4, Canda

^b^ Shandong Key Laboratory of Intelligent Energy Materials, School of Materials Science and Engineering, China University of Petroleum (East China), Qingdao, 266580, P.R. China

^c^ School of Petroleum Engineering, China University of Petroleum (East China), Qingdao 266580, P.R. China

* Corresponding authors: binghe@upc.edu.cn, luxq@upc.edu.cn, and b22140006@s.upc.edu.cn

**Materials**

The chemicals of Nickel (II) nitrate hexahydrate Ni(NO_3_)_2_·6H_2_O, Iron(Ⅲ) nitrate nonahydrate Fe(NO_3_)_3_·9H_2_O, Selenium Dioxide (SeO_2_), Hydrazine hydrate (N_2_H_4_·H_2_O), Glucose (C_6_H_12_O_6_), Potassium hydroxide (KOH, 85%), and Sodium chloride (NaCl, 99.5%) were used as received without any further purification treatment. The natural seawater was collected from Lingshan Bay, Huanghai Sea.

**Preparation of FeNi_2_Se_4_**

A homogeneous solution A was prepared by dissolving 1 mmol Fe(NO_3_)_3_·9H_2_O (99.99%, Aladdin) and 2 mmol Ni(NO_3_)_2_·6H_2_O (99.99%, Aladdin) in 20 mL deionized water under vigorous stirring. Solution B was prepared by dissolving 4 mmol SeO_2_ (99.99%, Aladdin) in 10 mL deionized water with 30 min of vigorous stirring, followed by dropwise addition of 2 mL N_2_H_4_·H_2_O (98.0%, Aladdin) and continued stirring for 30 min.

Solution A was rapidly added into solution B. The mixed solution was stirred for 30 min and transferred to a Teflon-lined autoclave. Hydrothermal treatment was conducted at 150 ℃ for 6 h. After cooling naturally to room temperature, the product was collected, sequentially washed with deionized water and absolute ethanol, and dried at 60 ℃ for 12 h.

**Preparation of C@FeNi_2_Se_4_**

The base synthesis process for FeNi_2_Se_4_ was maintained as previously described. To introduce C, varying masses of glugose were dissolved in Solution A prior to mixing with solution B. Samples were named as C_x_@FeNi_2_Se_4_ (where x= 0.5, 0.7, 0.9, 1.0, and 2.0 mmol), with the subscript x denoting the amount of glucose added during synthesis.

**Preparation of C spheres**

A solution of 0.9 mmol glugose dissolving in 40 mL deionized water was prepared under vigorous stirring. Then, the solution was transffered to a Teflon-lined autoclave. Hydrothermal treatment wasa conducted at at 150 ℃ for 6 h. After cooling naturally to room temperature, the product was collected before being washed with deionized water and absolute ethanol, and dried at 60 ℃ for 12 h.

**Material Characterization**

The composition of the samples was analyzed by X-ray diffraction (XRD) using a Philips X-Pert diffractometer (Holland, Cu Kα=1.540059 Å, operated at 40 kV). The morphology characterization was performed via Field emission scanning electron microscope (FESEM) on Hitachi S-480 equipped with an energy-dispersive X-ray spectrometer. Transmission electron microscopy (TEM) and high-resolution TEM (HRTEM) were conducted on a JEM-2100F microscope (JEOL, Japan). The surface composition of the electrode films was investigated using an X-ray photoelectron spectroscope (XPS) (a Thermo Fisher K-alpha 250 Xi). The inductively coupled plasma emission spectroscopy (ICP-MS) was investigated by Agilent 720ES.

**Electrochemical Measurements**

Electrochemical measurements were conducted at room temperature in 1 M KOH + 0.5 M NaCl and 6 M KOH + Seawater using a CHI 760E electrochemistry workstation. The electrochemical studies were carried out in a standard three-electrode system, with Pt foil and a Hg/HgO electrode serving as the counter electrode and reference electrode, respectively. The slope of the curves was calculated by the Tafel equation:

η = b*log (j/j_0_) (1)

The electrochemical impedance spectroscopy (EIS) analysis was performed in the frequency range of 10^5^-0.01 Hz. The stability of the catalyst was evaluated by applying DC power with Pt foil as the cathode in 1 M KOH + 0.5 M NaCl and 6 M KOH + Seawater. In a real seawater system, the stability was tested using a two-electrode system with Pt foil and C/NiFeOOH/SeO_4_^2-^ as the cathode and anode.

**Density functional theory (DFT) calculation**

The calculations were performed by spin-polarized density functional theory (DFT) as implemented in Vienna Ab initio Simulation Package (VASP) 6.1.0 with Perdew-Burke-Ernzerhof (PBE) generalized gradient approximation (GGA). The cutoff energy of 420 eV was selected after cutoff testing, and a 2*2*1 k-point grid was used for geometry optimization. The K-point for electronic densities of states calculation was set as 11*11*1. The 3d orbitals of Ni and Fe atoms were calculated for the active site on NiFeOOH, NiFeOOH/SeO_4_^2-^ and C/NiFeOOH/SeO_4_^2-^, respectively, in the calculation of densities of states. The electronic energy was converged to within 10^-5^ eV, and the forces were converged to within 0.02 eV/Å. The van der Waals interactions were considered by the method of the Grimme (DFT+D3). The effect of water was considered using the implicit solvent model in VASP.

Gibbs free energy changes were calculated by the computational hydrogen electrode (CHE) model. In this model, the reaction: H^+^_(aq)_ + e^-^ = 1/2 H_2(g)_ was equilibrated at 0 V vs the reversible hydrogen electrode at all pH values. The change of Gibbs free energy (△G) for each elementary step was defined as the following equation.

△G = △E + △E_ZPE_ - T△S + △G_U_ + △G_pH_ (2)

where △E represents the reaction energy, △E_ZPE_ and △S are the zero-point energy (ZPE) and the entropy difference between the products and the reactants at room temperature (T = 298.15 K), respectively. △G_U_ is the contribution of the applied electrode potential (U) to △G. and in this case, it is set as 0 V. The △G_pH_ denotes the free energy contribution resulting from variations in H concentration. However, in this work, the contribution of pH was not taken into account.

The OER could occur in the following elementary steps in alkaline conditions.

* + OH^-^ → *OH + e^-^ (3)

*OH + OH^-^ → *O + H_2_O + e^-^ (4)

*O + H_2_O → *OOH + e^-^ (5)

*OOH + OH^-^ →* + O_2_ + H_2_O + e^-^ (6)

Where * stands for an active site on the surface, *OH, *O, and *OOH are the adsorbed intermediates.


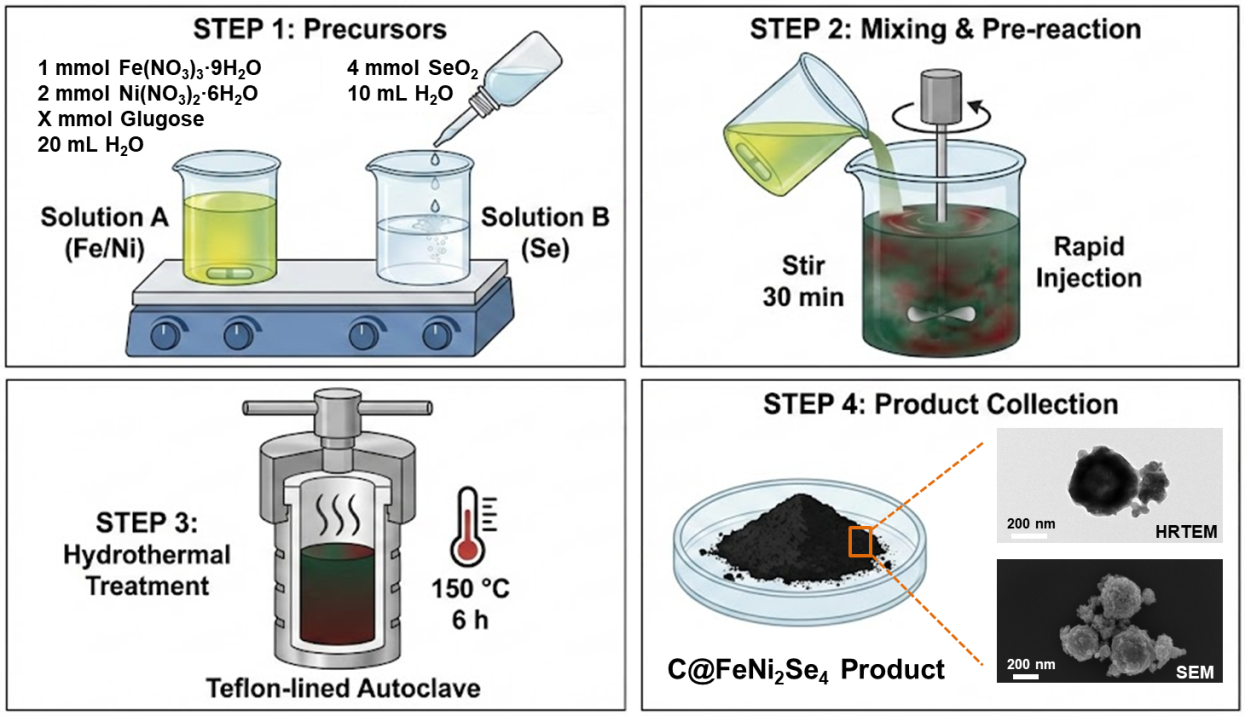


Figure S1 Diagram of preparation process of C@FeNi_2_Se_4_ catalyst.


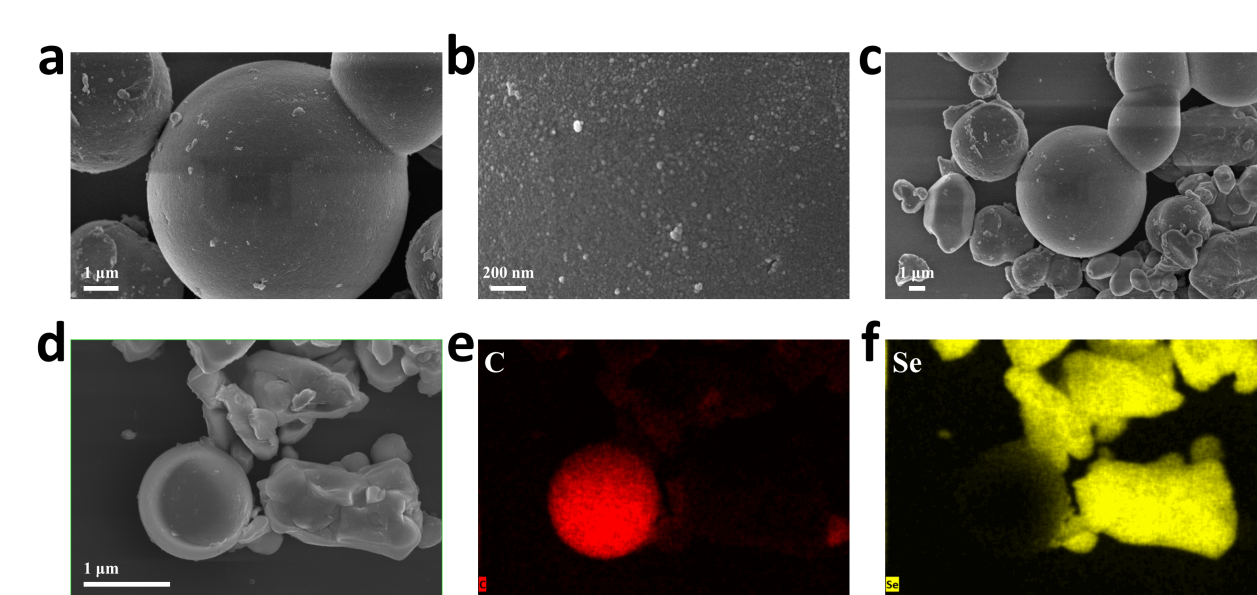


Figure S2. SEM images of C sphere and corresponding elemental mapping of C sphere.


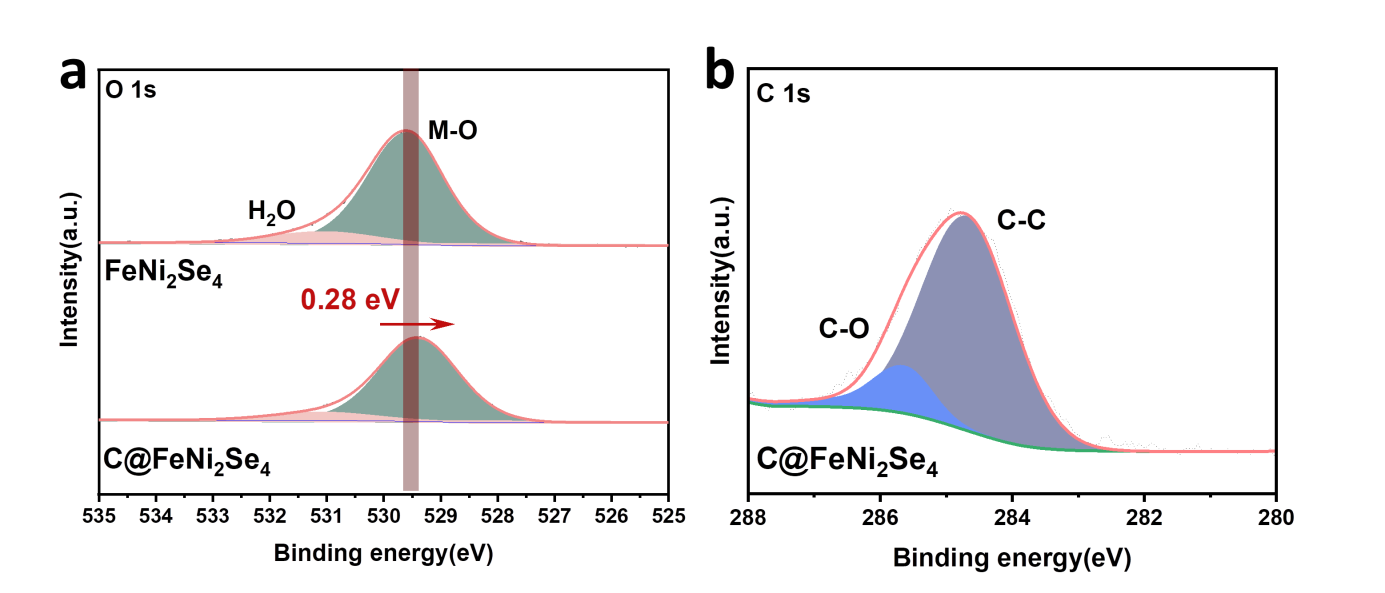


Figure S3. (a) High-Resolution XPS spectra of O 1s for FeNi_2_Se_4_ and C@FeNi_2_Se_4_. (b) High-Resolution XPS spectra of C 1s for C@FeNi_2_Se_4_.


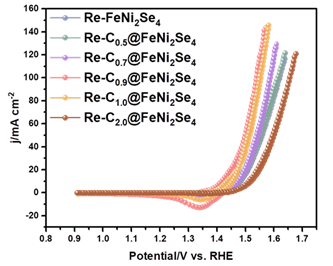


Figure S4. The CV curves for Re-FeNi_2_Se_4_, Re-C_0.5_@FeNi_2_Se_4_, Re- C_0.7_@FeNi_2_Se_4_, Re- C_0.9_@FeNi_2_Se_4_, Re-C_1.0_@FeNi_2_Se_4_, and Re-C_2.0_@FeNi_2_Se_4_.


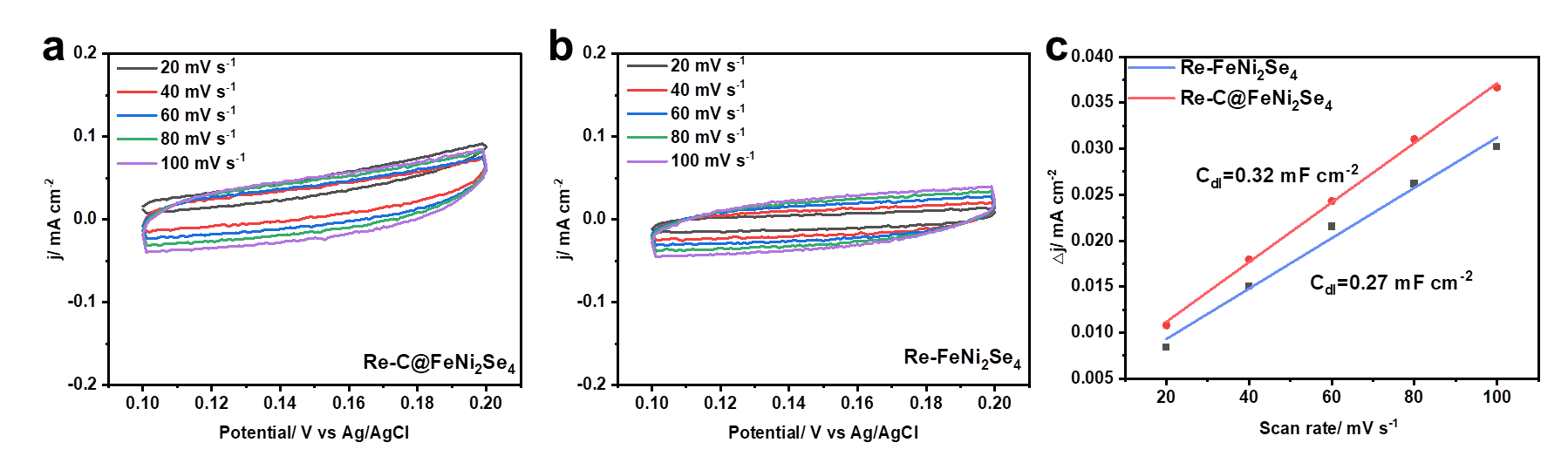


Figure S5. CV curves for (a) Re-C@FeNi_2_Se_4_ and (b) Re-FeNi_2_Se_4_ in the region of 0.10-0.20 V vs. Ag/AgCl with various scan rates for OER. (c) C_dl_ values of Re-FeNi_2_Se_4_ and Re-C@FeNi_2_Se_4_.


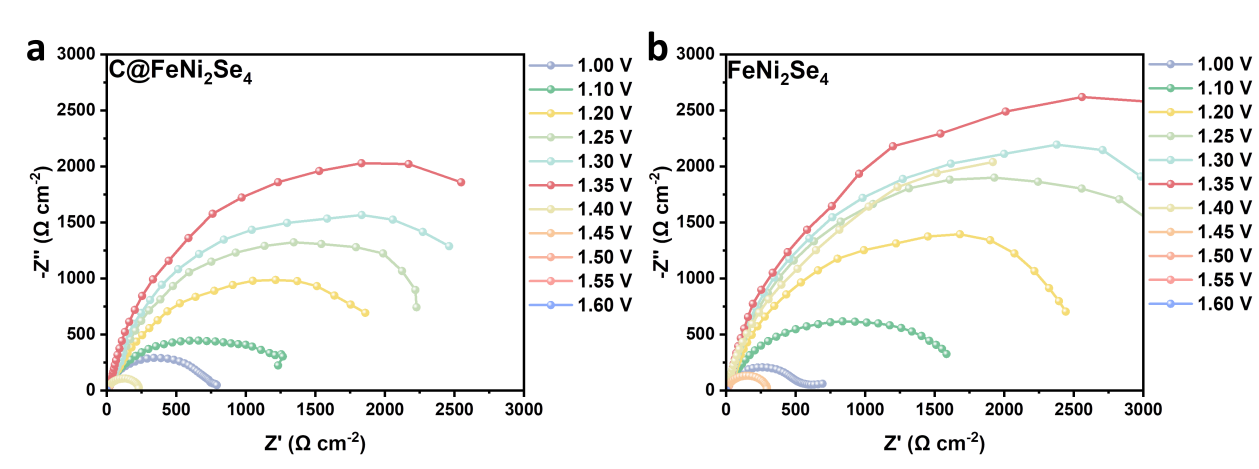


Figure S6. Nyquist of the EIS data for the (a) C@FeNi_2_Se_4_ and (b) FeNi_2_Se_4_ for OER at different potential in 1 M KOH + 0.5 M NaCl.


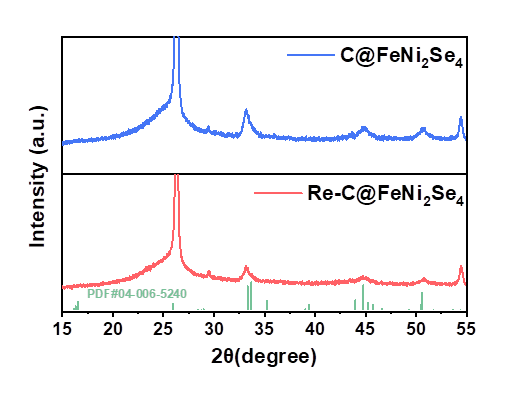


Figure S7 XRD patterns of C@FeNi_2_Se_4_/C paper and Re-C@FeNi_2_Se_4_/C paper.





Figure S8 In situ Raman spectra of C@FeNi_2_Se_4_ in 1 M KOH + 0.5 M NaCl.


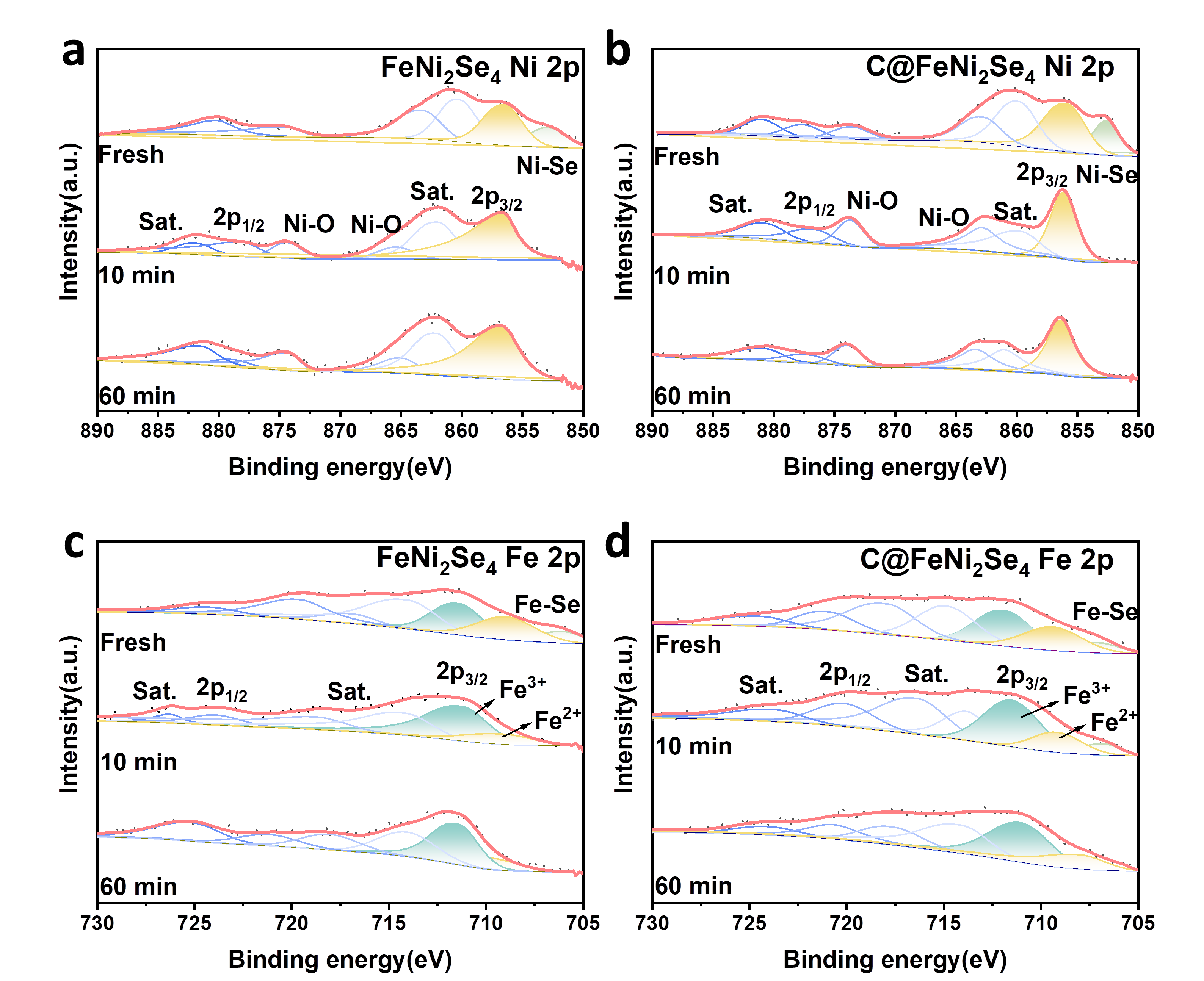


Figure S9 XPS spectra of Ni 2p for FeNi_2_Se_4_ (a) and C@FeNi_2_Se_4_ (b) after 10 and 60 min of OER at 100 mA cm-^2^. XPS spectra of Fe 2p for FeNi_2_Se_4_ (c) and C@ FeNi_2_Se_4_ (d) after 10 and 60 min of OER at 100 mA cm^-2^


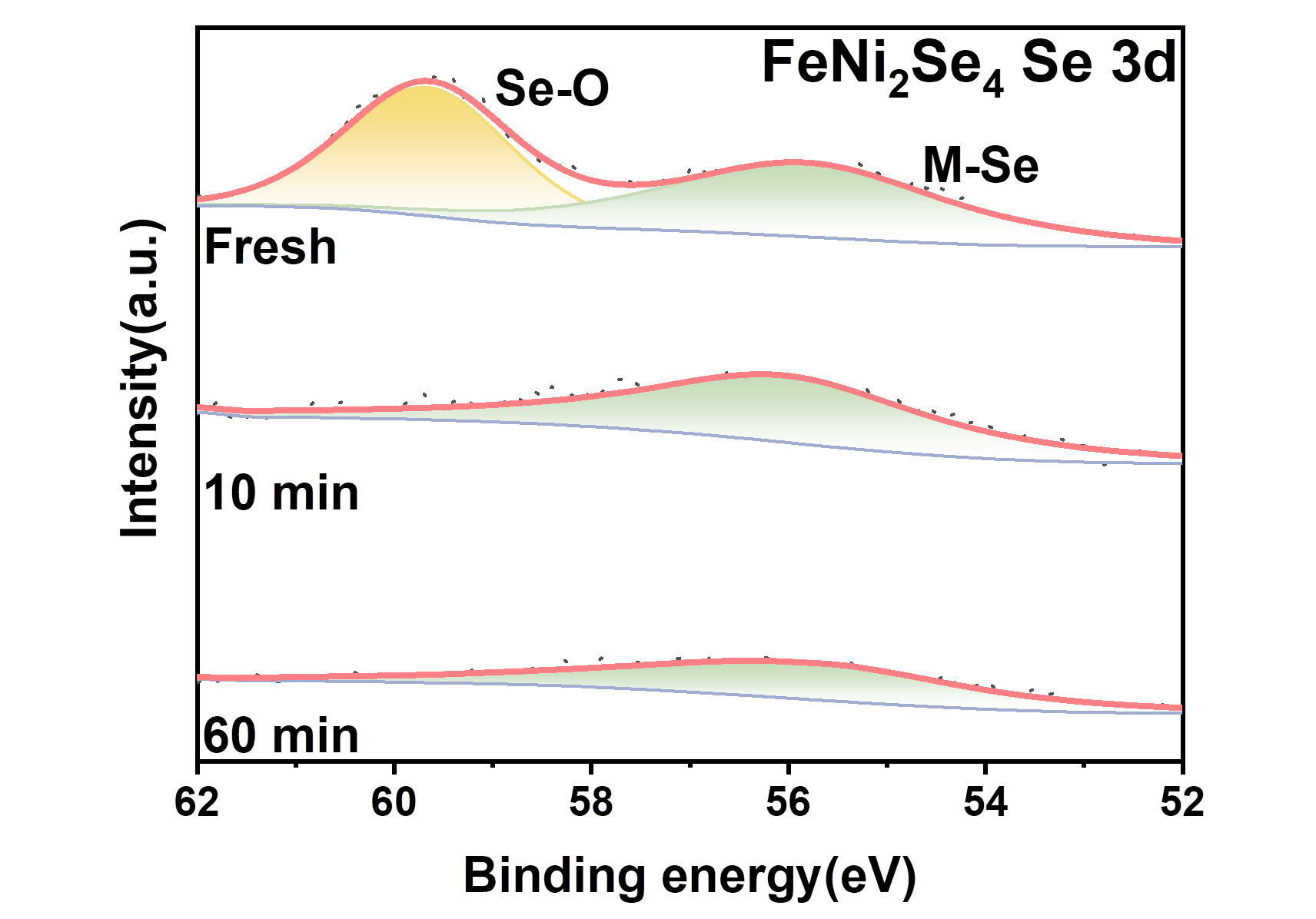


Figure S10 XPS spectra of and Se 3d (c) for FeNi_2_Se_4_ after 10 and 60 min of OER at 100 mA cm^-2^.


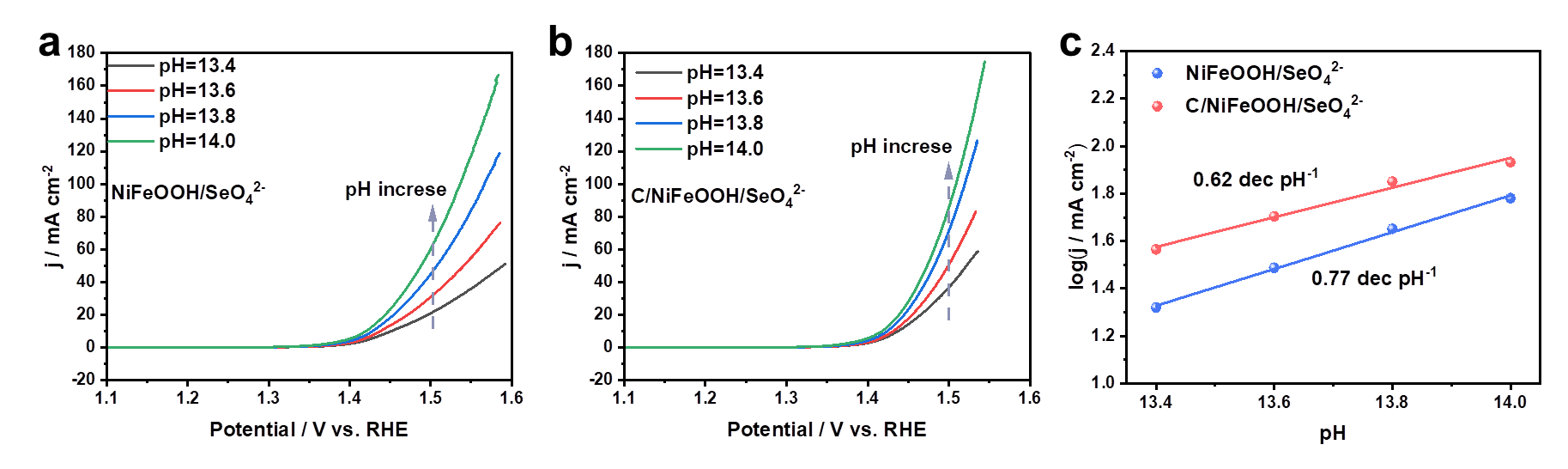


Figure S11 LSV curves of NiFeOOH/SeO_4_^2-^ (a) and C/NiFeOOH/SeO_4_^2-^ (b) in KOH electrolytes with different pH values. (c) The relationship between the log (j) at the potential of 1.5 V vs. RHE and different pH values.


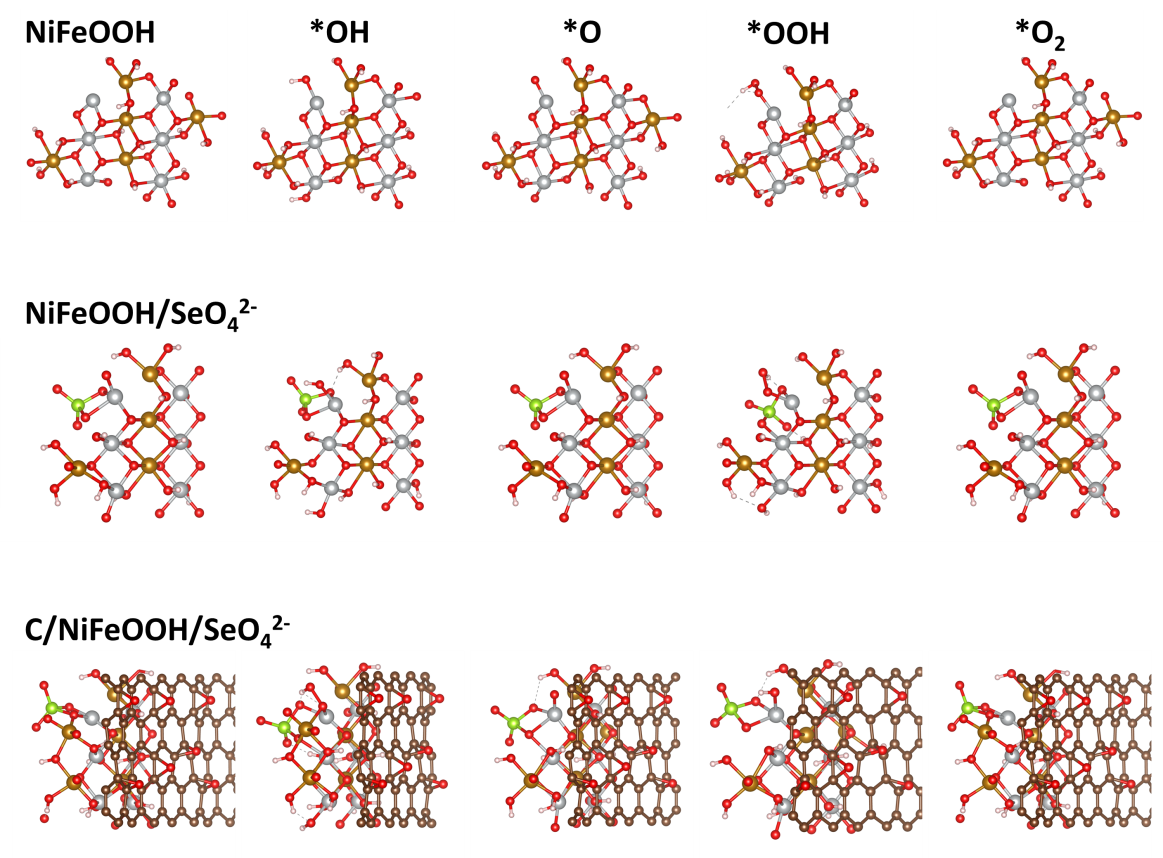


Figure S12 Optimized structure and OER pathway of NiFeOOH, NiFeOOH/SeO_4_^2-^, and C/NiFeOOH/SeO_4_^2-^.


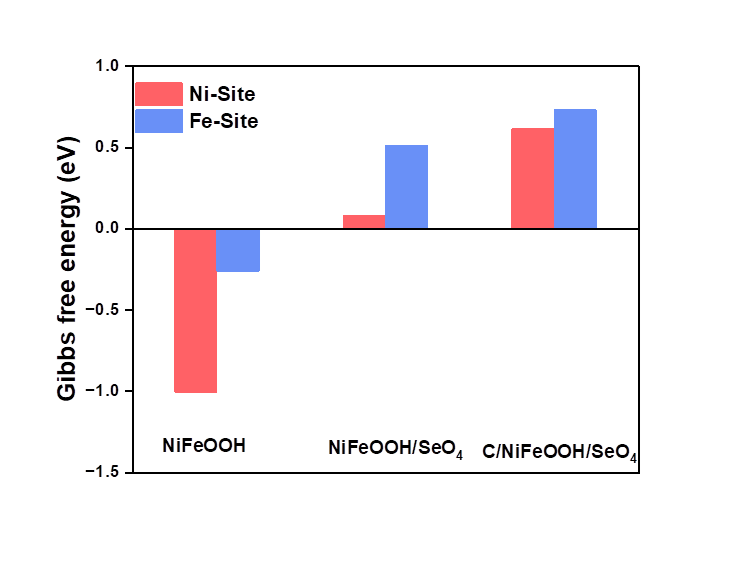


Figure S13 The Gibbs free energy of *OH on both Ni and Fe centers of NiFeOOH, NiFeOOH/SeO_4_^2-^, and C/NiFeOOH/SeO_4_^2-^.


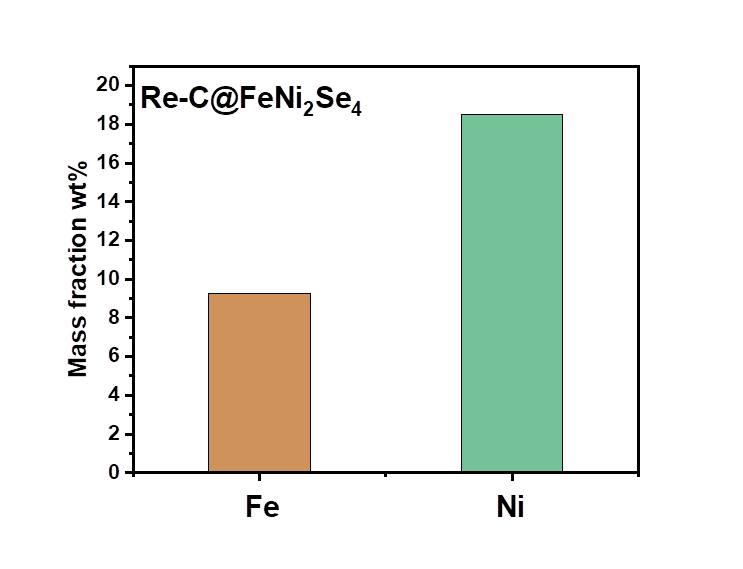


Figure S14 Mass fraction of C/NiFeOOH/SeO_4_^2-^ by ICP test.


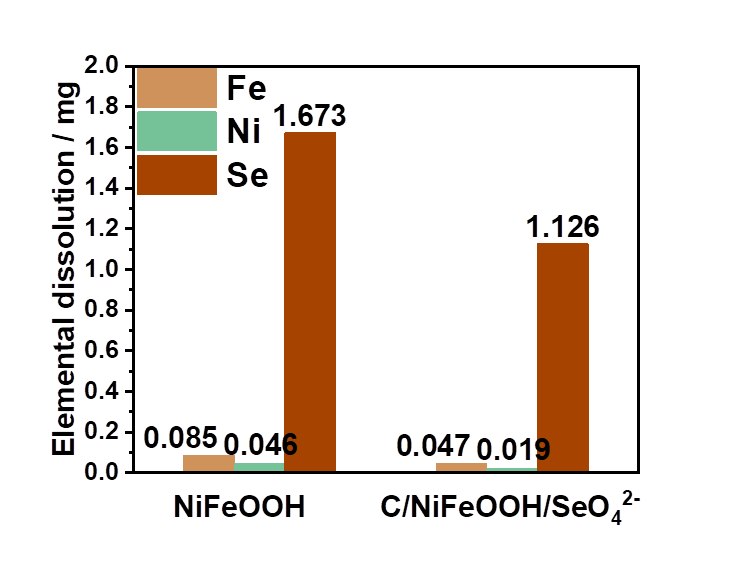


Figure S15 The dissolution of Ni, Fe, and Se of NiFeOOH and C/NiFeOOH/SeO_4_^2-^ after stability test in 1 M KOH + Seawater under 200 mA cm^-2^.

Table S1 Comparison of OER performance with other recently reported electrocatalysts.

| **Sample** | **Electrolyte** | **Overpotential**  **mV (10 mA cm^-2^)** | **Tafel Slope**  **(mV dec^-1^)** | | **Durability**  **(h)/(** **mA cm^-2^)** |
| --- | --- | --- | --- | --- | --- |
| This work | 1 M KOH + 0.5M NaCl | 223 | | 39.4 | 150/400 |
| NiFeP/Ni_3_S_2_ | 1 M KOH + 0.5M NaCl | 256 | | 36.1 | 120/100 |
| Ni_3_FeN@PO_4_^3−^/NF | 1 M KOH + 0.5M NaCl | 238 | | 29.9 | 2500/1000 |
| Sn-NiFeOOH@NF | 1 M KOH + 0.5M NaCl | 272 | | 63.6 | 200/600 |
| S-NiCoP | 1 M KOH + 0.5M NaCl | 236 | | 118 | 200/10 |
| NC-CoNi_2_S_4_@ReS_2_/CC | 1 M KOH + 0.5M NaCl | 253 | | 54.7 | 60/100 |
| NiCoMoPO | 1 M KOH + 0.5M NaCl | 272 | | 67.6 | 300/1000 |
| WO_3_/CoP@NF | 1 M KOH + 0.5M NaCl | 343 | | 60 | 100/100 |
| 2-CNFMX | 1 M KOH + 0.5M NaCl | 240 | | 55 | 60/200 |

Table S2. Fitting parameters for the impedance date of FeNi_2_Se_4_ in 1M KOH + 0.5 M NaCl.

| Potential/V | RS | C1 | R1 | C2 | R2 |
| --- | --- | --- | --- | --- | --- |
| 1.00 | 1.410 | 0.00013 | 493.10 | 0.00010 | 493.10 |
| 1.10 | 1.407 | 0.00042 | 969.50 | 0.00174 | 848.80 |
| 1.20 | 1.400 | 0.00269 | 36.09 | 0.00062 | 3048.00 |
| 1.25 | 1.410 | 0.00082 | 1.36 | 0.00082 | 5823.00 |
| 1.30 | 1.379 | 0.00237 | 29.98 | 0.00096 | 4899.00 |
| 1.35 | 1.372 | 0.00238 | 25.65 | 0.00110 | 6096.00 |
| 1.40 | 1.372 | 0.00167 | 4794.00 | 0.00575 | 13.97 |
| 1.45 | 1.360 | 0.00330 | 286.50 | 0.01202 | 9.84 |
| 1.50 | 1.355 | 0.00611 | 3.76 | 0.01391 | 6.40 |
| 1.55 | 1.370 | 0.00385 | 1.54 | 0.12730 | 0.42 |
| 1.60 | 1.403 | 3.46800 | 0.17 | 0.00397 | 0.70 |

Table S3. Fitting parameters for the impedance date of C@FeNi_2_Se_4_ in 1M KOH + 0.5 M NaCl.

| Potential/V | RS | C1 | R1 | C2 | R2 |
| --- | --- | --- | --- | --- | --- |
| 1.00 | 1.436 | 0.00088 | 119.50 | 0.00011 | 688.40 |
| 1.10 | 1.463 | 0.00160 | 756.00 | 0.00026 | 715.20 |
| 1.20 | 1.457 | 0.00357 | 10.30 | 0.00060 | 2587.00 |
| 1.25 | 1.438 | 0.00200 | 75.06 | 0.00081 | 2900.00 |
| 1.30 | 1.429 | 0.00241 | 35.94 | 0.00098 | 3684.00 |
| 1.35 | 1.420 | 0.00117 | 4811.00 | 0.00216 | 31.07 |
| 1.40 | 1.407 | 0.00348 | 226.70 | 0.01056 | 10.93 |
| 1.45 | 1.400 | 0.02248 | 6.19 | 0.00599 | 3.49 |
| 1.50 | 1.413 | 0.12140 | 0.60 | 0.00398 | 1.61 |
| 1.55 | 1.473 | 0.00325 | 0.79 | 0.82950 | 0.20 |
| 1.60 | 1.503 | 0.00228 | 0.32 | 2.93500 | 0.19 |
